# Unraveling Synthetase's Mode of Action: The Pyrrolysyl‐tRNA Synthetase Dimer Uses Secondary Binding Sites in the Cell

**DOI:** 10.1002/anie.202514065

**Published:** 2026-03-24

**Authors:** Jessica Dröden, Christoph Globisch, Eliane Landwehr, Theresa S. Braun, Daniel Summerer, Christine Peter, Malte Drescher

**Affiliations:** ^1^ Department of Chemistry and Konstanz Research School Chemical Biology University of Konstanz Konstanz Germany; ^2^ Faculty of Chemistry and Chemical Biology TU Dortmund University Dortmund Germany; ^3^ RPTU University Kaiserslautern‐Landau Kaiserslautern Germany

**Keywords:** enzyme catalysis, EPR spectroscopy, molecular dynamics simulation, substrate‐ligand interaction

## Abstract

Aminoacyl‐tRNA synthetases mediate the activation and transfer of amino acids to their cognate tRNA, which constitutes one of the initial events in protein biosynthesis. Even though different mechanisms of action have been proposed for the catalysis of these enzymes, their entire catalytic cycle remains elusive. Here, we used electron paramagnetic resonance spectroscopy in vitro and in cells in combination with molecular dynamics simulations to study the role of amino acid interactions in the catalytic cycle of pyrrolysyl‐tRNA synthetases (PylRS), a widely used tool for genetic code expansion. Experiments using the paramagnetic non‐canonical amino acid SLK‐1 revealed the presence and occupation of secondary amino acid binding sites in PylRS located at the intermonomer interface, distant from the catalytic binding site. Based on our results, we propose a model that assumes an alternating mode of action of the two PylRS monomers for the catalytic cycle of PylRS.

## Graphical abstract

The pyrrolysyl‐tRNA synthetase dimer exhibits an alternating mode of action of its monomers within its catalytic cycle, which is realized upon occupation of secondary binding sites located at the intermonomer interfaces outside the catalytic binding site.

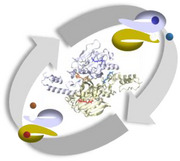

One of the initial events in protein biosynthesis is the selective activation and transfer of amino acids to their cognate tRNA. This step is mediated by aminoacyl‐tRNA synthetase (aaRS) enzymes [1, 2, 3]. Depending on the structure and fold of their catalytic domain, aaRSs are divided into two different main classes, named class I and II, that exhibit different modes of substrate binding [4, 5]. Previous studies focusing on the mechanisms of the activation and transfer reaction in molecular detail relied largely on kinetic assays [6]. Important approaches have included pyrophosphate exchange, aminoacylation assay, stopped‐flow fluorescence for investigating substrate binding rates and affinities, and the formation of intermediates to determine the underlying catalytic cycle [7]. Based on these results, different mechanisms for the reaction have been proposed, but the data is still insufficient to clearly articulate the entire catalytic cycle.

A prominent aaRS example is the *M. mazei* tRNA^Pyl^/ pyrrolysyl‐tRNA synthetase (PylRS) pair [8, 9], which is frequently used to genetically encode a variety of non‐canonical amino acids in different organisms [10, 11, 12, 13]. In‐depth knowledge about its mechanism would increase its applicability for incorporating new chemical and biophysical functions into proteins to study their structure and function in vitro and in vivo [14, 15]. PylRS is a class IIc aaRS enzyme, which exists as an obligate homodimer. The C‐terminal catalytic domain (amino acid residues 185–454) exhibits the typical seven‐stranded β‐sheet fold, which allows it to bury its amino acid substrate within a deep hydrophobic binding pocket [16, 17]. Structures of the catalytic domain in complex with a series of non‐canonical amino acids (ncAAs) were solved by x‐ray crystallography, which greatly contributed to understanding the molecular underpinnings of ncAA binding in the amino acid binding pocket of PylRS [18, 19]. However, crystal structures might not reflect all physiologically relevant or metastable synthetase conformations or amino acid binding modes, nor provide information about transient steps in the catalytic cycle.

In this study, we used the paramagnetic amino acid SLK‐1 [20, 21] (Figure 1A) to investigate the interaction of ncAAs with the catalytic domain (cd) of a PylRS proficient in activating and charging this amino acid via electron paramagnetic resonance (EPR) spectroscopy [22, 23, 24, 25]. While cdPylRS is more easily accessible in experiments, we extended our approach also to full‐length PylRS. This strategy enables studying amino acid interactions of PylRS in the non‐crystalline state, including studies of the fully functional tRNA^Pyl^/ PylRS pair expressed in *Escherichia coli* cells. By combining double electron–electron resonance (DEER) distance measurements with molecular dynamics simulations, we discovered a new amino acid binding site at the PylRS dimer interface, which provides clues to previously unknown steps in the catalytic cycle of PylRS.

**FIGURE 1:**
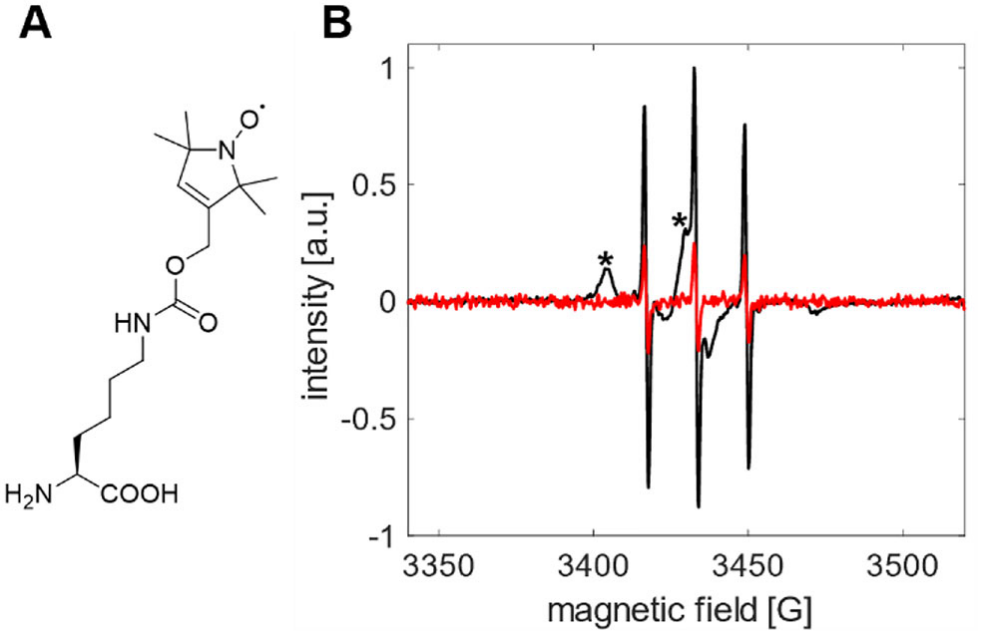
The binding of SLK‐1 to cdPylRS‐SL1 in vitro studied by EPR experiments. (A) Chemical structure of the ncAA SLK‐1. (B) Room‐temperature EPR spectra of SLK‐1 in the presence of cdPylRS‐SL1 in vitro (black) or cdPylRS (Y306A/ Y384F) in vitro (red), initial dimer.SLK‐1 ratio of 1:15, spectra were recorded upon the washing procedure (see Supporting Information). A broadened spectrum of cdPylRS‐SL1 (asterisks) reveals binding of SLK‐1, whereas cdPylRS lacking the I413L mutation was not able to bind SLK‐1.

First, we set out to characterize SLK‐1 binding to cdPylRS in vitro. For our experiments, we used the variant cdPylRS‐SL1 [20], which contains only a single conservative I413L mutation in combination with the mutations Y306A and Y384F (all in the PylRS substrate binding pocket). The latter two mutations result in high activity and broad substrate tolerance for bulky ncAA, which has led to their wide use for the encoding of ncAA [10, 11]. This makes cdPylRS‐SL1 an ideal model for studying mechanisms of PylRS with high application relevance. Like PylRS [16, 17], purified cdPylRS‐SL1 forms dimers to a large extent (Figure S1). To demonstrate the binding of SLK‐1, we incubated purified cdPylRS‐SL1 with SLK‐1 and measured EPR spectra (Figures 1B, S2, and S3). The EPR spectrum of SLK‐1 directly correlates with its rotational diffusion. Spectral broadening due to slower diffusion indicates the binding of SLK‐1 to its target. The broad component revealed binding of SLK‐1 at cdPylRS‐SL1 in a very rigid state. Besides the broad component, the sharp peaks in the spectrum showed the presence of unbound SLK‐1 (9%, derived by spectral simulations, Figure S3). The concentration‐dependent EPR data (Figure S2) suggest affinities of SLK‐1 in the high micromolar range. It is worth mentioning that at this affinity we find the complete bio‐orthogonal expression system to be functional (see below). No significant SLK‐1 binding was observed when the I413L mutation in cdPylRS (refers to PylRS Y306A/ Y384F variant throughout the manuscript) was absent (Figure 1B), highlighting the importance of this modification for SLK‐1 binding.

To predict the binding mode of SLK‐1 to cdPylRS‐SL1, we performed docking studies and molecular dynamics (MD) simulations. As an initial structure, we used the reported structure in complex with (2R)‐2‐amino‐6‐[(phenylmethoxy)carbonylamino]‐4‐selena‐hexanoic acid (pdb ID: 6AAP [19]) due to the presence of the Y306A and Y384F mutation and the great similarity of the amino acid substrates. We introduced the I to L mutation at position 413, chose two alternative positions for the leucine side chain according to the branching of the isoleucine side chain, which resulted in two models, L1 and L2, and docked SLK‐1 into the catalytic binding sites a.1 and b.1 of the cdPylRS‐SL1 dimer (Figure 2). As expected, we found SLK‐1 positioned within the two hydrophobic binding sites, which places the nitroxide moiety near the I413L mutation (Figure S4). To verify the quality of the obtained docking poses and investigate the stability of SLK‐1 in the binding sites, 500 ns long MD simulations were started from the three best‐scored representative poses of each model, L1 and L2. Analysis of the binding stability of the two SLK‐1 molecules during the simulations suggests an alternating binding of the ligand to the two catalytic binding sites. In all the simulations, one of the two ligands showed a larger displacement from the initial docked position while the other stayed in place (see the distributions of the ligand displacement of the weak and strong binding species in Figure S5).

**FIGURE 2:**
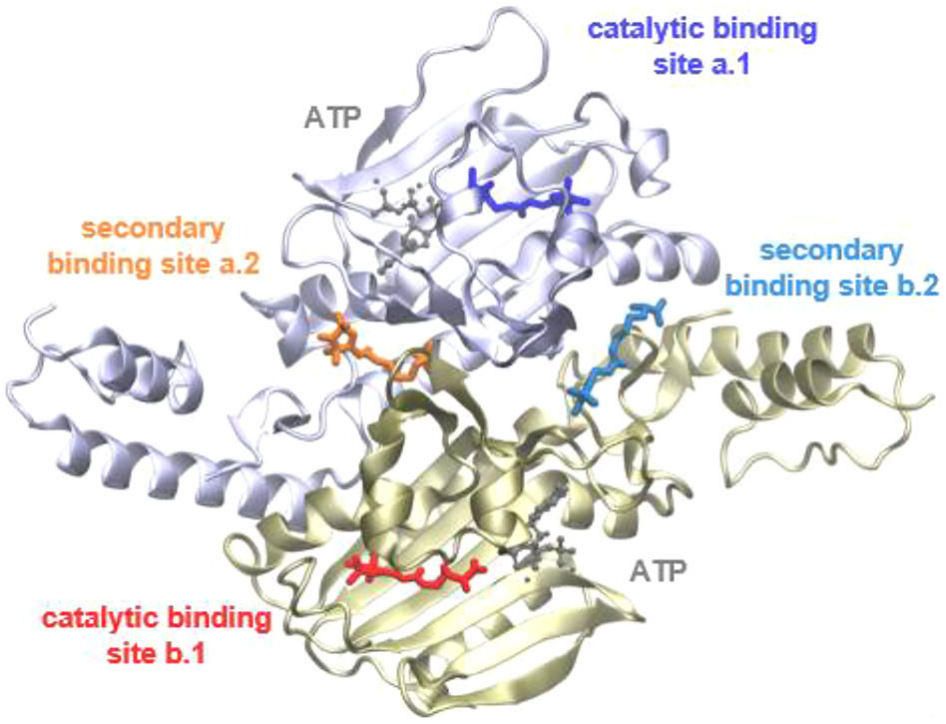
Binding sites of cdPylRS‐SL1. The dimeric structure of cdPylRS‐SL1 (based on pdb ID: 6AAP [19]) in complex with ATP and SLK‐1, with the four different binding sites indicated as derived from docking experiments.

Interestingly, when repeating the docking into cdPylRS‐SL1 with a wider radius around the catalytic binding sites, we found two secondary binding positions per dimer (secondary binding sites a.2 and b.2) located at the intermonomer interface. For the structures docked into the secondary binding sites, a.2 and b.2 (Figure 2), we observed lower scores compared with the catalytic binding sites, which indicates a weaker binding. This observation was supported by MD simulations with the docked poses starting from the x‐ray structure, leading to predominantly short residence times (far below 100 ns) in the secondary binding sites (Figure S6).

To unravel the binding mechanism of SLK‐1 to cdPylRS‐SL1 experimentally, we performed DEER experiments, also named PELDOR (pulsed electron‐electron double resonance), to determine the distance distributions between two paramagnetic centers in the nanometer range [26, 27, 28, 29, 30, 31]. We incubated SLK‐1 with purified cdPylRS and cdPylRS‐SL1, respectively, applied the washing procedure (see Supporting Information), and performed the DEER experiment (Figures 3A,B and S7). For cdPylRS lacking the I413L mutation, we expected SLK‐1 not to bind. Indeed, the signal intensity was very low (Figure S8). For cdPylRS‐SL1, the obtained DEER trace showed clear oscillations, which indicate a well‐defined distance at 2.5 nm. The narrow distribution corroborated the finding that SLK‐1 is very rigidly bound to PylRS‐SL1. We have excluded orientation selectivity by varying the spectral positions of the DEER pulses without monitoring changes in the shape of the DEER trace (Figure S19).

**FIGURE 3:**
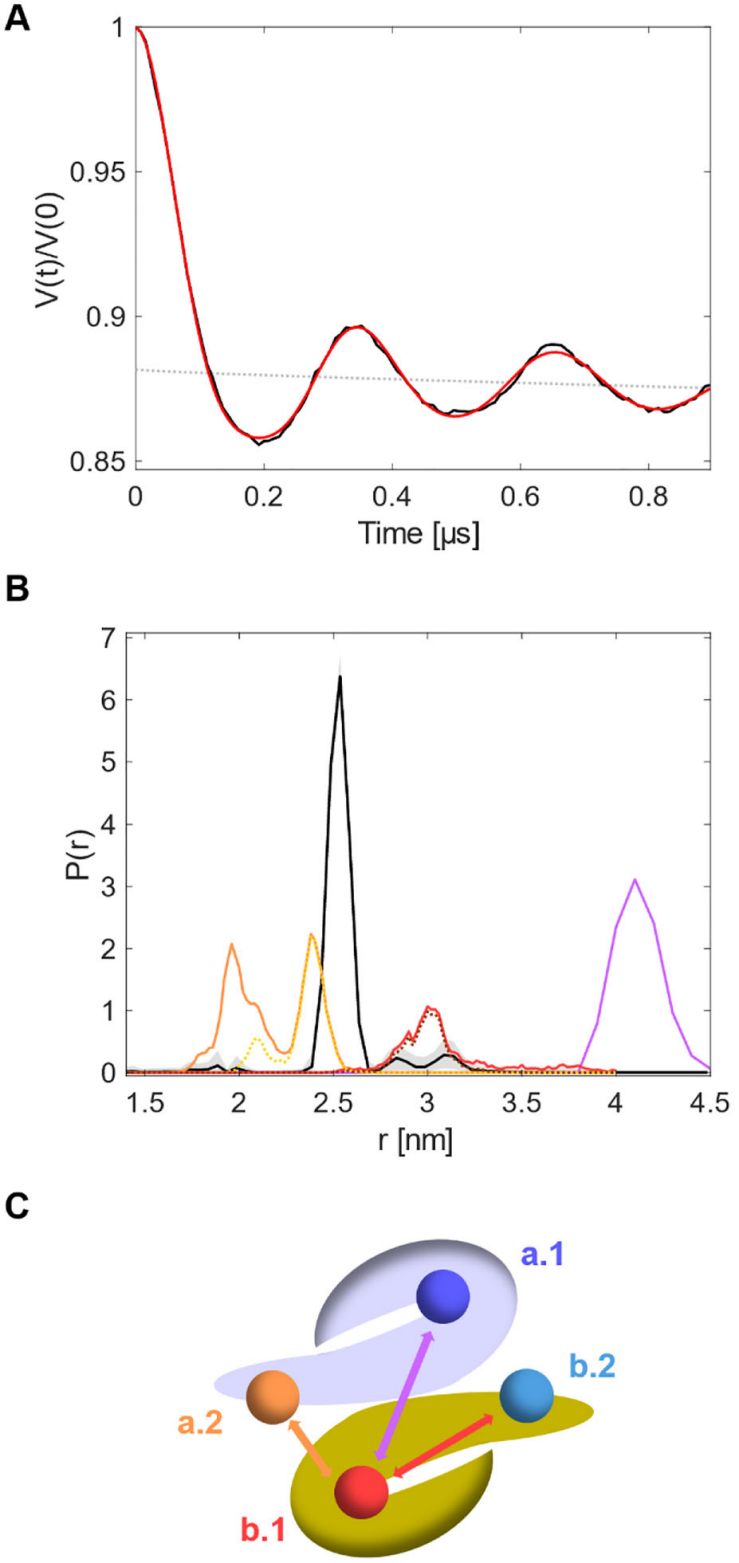
DEER measurements of SLK‐1 in the presence of cdPylRS‐SL1 in vitro. (A) DEER raw data with background (gray) and fit (red). (B) Comparison of experimental consensus distance distribution (black) with simulated distance distributions (colored) of distances between SLK‐1 pairs occupying three different combinations of binding sites (colored lines, the color code corresponds to the pairings indicated in (C)). The gray‐shaded area indicates the uncertainty. The dotted distance distributions (yellow, brown) represent the probability distributions after accounting for the residencies of the ligands in the secondary binding sites in the different simulations. For details, see the method section. (C) Schematic representation of cdPylRS‐SL1 dimer and the different binding sites occupied with SLK‐1. (For the equivalent distances between the secondary binding sites and a.1 see Figure S9, in our experiments, we would expect a corresponding superposition.

Analysis of the modulation depth Δ and the signal intensity of EPR spectra revealed that 9% of all PylRS‐SL1 dimers are doubly occupied, 21% are singly occupied, and 70% are without a ligand under the applied conditions. The fact that more doubly occupied dimers are found than expected for a random distribution of SLK‐1 over the binding sites indicates a sort of cooperative binding of SLK‐1 to the binding sites of PylRS‐SL1.

DEER is a powerful method for determining full distance distributions with respective probabilities, rather than just mean distances. It can thus be used to investigate complex binding scenarios [32]. Here, analysis of the DEER trace resulted in a single narrow distance peak at 2.5 nm, indicating that all the possible binding sites of cdPylRS‐SL1 were not occupied at the same time. Also, the phylogenetically closest aaRS, phenylalanyl‐tRNA synthetase (PheRS), was found to be bound by two substrates at high amino acid concentrations in early studies [33, 34].

To identify the occupied binding sites, we overlaid the experimental distance distribution (Figure 3B, black) with distance distributions calculated from MD simulations. We clustered the combined trajectories of the first set of MD simulations with ligands only bound to the catalytic binding sites and repeated the docking procedure for the secondary binding sites with representative protein structures from the clustering. This improved the docking scores, and we selected the protein structures that scored well with stable binding in both the catalytic and the secondary binding sites. From these structures, further MD simulations were carried out to predict distance distributions between the paramagnetic moieties of SLK‐1 for different pairs of potentially occupied binding sites (Figure 3B,C [orange, red, purple] and Figure S9). We do not consider the case of simultaneous and exclusive occupation of both secondary sites, a.2 and b.2, because when the catalytic binding sites, a.1 and b.1, of cdPylRS lacking the I413L mutation are not available for binding, the secondary sites, a.2 and b.2, do not play a role either (Figure 1B). From the experimental distance distribution, we could directly exclude one of the most obvious hypotheses: we did not find both catalytic binding sites, a.1 and b.1, occupied at the same time, which would have resulted in a distance of 4.1 nm (Figure 3B,C [purple]). We concluded that the binding pattern that corresponds to the presence of substrates in both catalytic binding sites, as observed in x‐ray crystallography, may originate from the experimental conditions (e.g., very high ligand concentrations) or other environmental effects in the crystal [16, 17]. Since average DEER distances from predictions typically correspond well with experimental values within a few Ångström [35] the data suggests that there is no occupation of both binding sites within one monomer of the dimer, for example, a.1 and a.2 *or* b.1 and b.2, because the predicted distance seems too long (∼ 3 nm, Figure 3B,C [red]) to fit the experimental result.

Finally, the binding pattern involving one catalytic binding site and one of the secondary binding sites, for example, SLK‐1 binding to sites a.1 and b.2 *or* b.1 and a.2 (Figure 3B,C [orange] and Figure S9 [light blue]) was in agreement with the experimental data. While the MD simulations started from the docked structures did in general support the hypothesis of the secondary binding sites a.2 and b.2, the binding is (expectedly) not particularly tight. In consequence, it was not possible to obtain well equilibrated, that is, quantitative, data regarding the occupation of the secondary binding sites and the distance distributions involving these sites in Figures 3B and S9.

Motivated by the observation of several rebinding attempts of the ligand to the secondary binding sites, we conducted a further set of simulations with an excess of free ligand in solution to increase the chance of binding. Here, multiple binding events to the—initially empty—secondary binding sites were observed where the ligand remains bound for extended periods of simulation time. This further supports the postulation of this secondary binding site. The respective data and a more detailed discussion can be found in Figures S10 and S11. We would like to use these data also to explicitly point out that the crystal structure is, in general, an excellent representative also for the structure of the protein in solution. No major structural changes are found. It is difficult to assess at this point why no occupation of the secondary binding sites has been found in the crystal structure, but given the relatively weak binding, quite subtle effects of crystallization conditions or the local environment in the protein crystal may easily be at play here. We thus obtained experimental evidence that the secondary binding sites a.2 and b.2 are indeed occupied in vitro.

Next, we investigated whether the secondary binding sites found in vitro are also populated in the cell. EPR spectroscopy in the cell has successfully been applied to investigate proteins directly inside cells [36, 37, 38, 39, 40, 41], even at nanomolar protein concentration [42]. There is a paramagnetic Manganese background signal in E. coli cells (Figure S15), which influences the modulation depth of Nitroxide‐Nitroxide‐DEER. Here, we expressed cdPylRS‐SL1, as well as the tRNA^Pyl^/ full‐length PylRS‐SL1 pair in the presence of SLK‐1 in *E. coli* cells. In the latter case, the whole functional machinery was present and able to incorporate SLK‐1 into a target protein (Figure S12). Again, analyzing spectral broadening in EPR spectra enabled us to show the binding of SLK‐1 to PylRS‐SL1 inside the cell (Figure S13). In control experiments, we excluded that this spectral broadening arises from the binding of SLK‐1 to other cellular components (i.e., when PylRS‐SL1 was absent; Figure S14).

We next performed in‐cell DEER experiments to investigate the intracellular binding pattern (Figures 4 and S15–S18). We found similar traces compared to the in vitro experiments; however, the length of the dipolar in‐cell trace (1.5 µs, Figure S17) defines the longest distance reached for this study in the cell to about 4 nm [28]. Within this range, we monitor the identical narrow distance distributions that correspond to a single distance at 2.5 nm (Figures 4, S16, and S17). In this case, comparing the occupation ratio with the in vitro results was not possible due to the limited stability of the nitroxide in the reductive cellular environment, which resulted in low modulation depths [43, 44]. In any case, we found the same distance of 2.5 nm in the cell and thus the same binding pattern, that is, a.1 and b.2 *or* b.1 and a.2, as found in vitro. We have thus provided the first experiment demonstrating the binding of a substrate to aaRS under physiologically relevant conditions.

**FIGURE 4:**
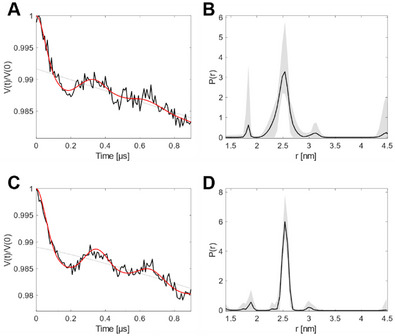
In‐cell DEER measurements of SLK‐1. (A) DEER raw data with background (gray) and fit (red) for the (B) consensus distance distribution of cells expressing cdPylRS‐SL1. (C) DEER raw data with background (gray) and fit (red) for the (D) consensus distance distribution of cells expressing full‐length PylRS‐SL1. Gray‐shaded areas present uncertainty. For the detected dipolar evolution time of ∼0.8 µs, only distances at approximately 3 nm or below yield reliable distance information [28], DEER data with a longer dipolar evolution time are shown in Figure S17.

Expanding the genetic code with ncAA has enabled the site‐specific incorporation of a large variety of chemical and biophysical functions into proteins in vitro and in vivo. This approach relies on the use of designed orthogonal pairs of tRNA and aaRS, with the *M. mazei* tRNA^Pyl^/ PylRS pair being a particularly widely used example [45]. X‐ray studies have focused nearly exclusively on understanding the binding of ncAA in the aminoacylation active site of PylRS, but protein crystals do not necessarily reflect physiologically relevant conformations and binding modes of substrates. We studied PylRS‐SL1 in solution with EPR spectroscopy by using the paramagnetic ncAA SLK‐1. In addition to the commonly known, deep binding pockets of the aminoacylation sites reported in the literature [16, 17], our MD simulations predicted two secondary binding sites per dimer with lower binding stability. In 1975, Fersht et al. speculated that monomeric aaRS had the ability to bind two substrate amino acids, hinting at the existence of secondary sites in addition to catalytically active ones [46]. However, information on their location has been missing.

DEER experiments demonstrated the binding of two SLK‐1 molecules per PylRS‐SL1 dimer. The binding of two substrate molecules with different affinities has also been reported for the phylogenetically closest aaRS, PheRS [33, 34, 47]. In our study, comparing experimental distance distributions with predicted distance distributions from MD simulations suggested that one ncAA molecule is placed within the catalytic binding site, whereas the other ncAA is located at the secondary binding site of the other PylRS monomer, indicating intermolecular cooperativity, meaning, that both subunits are required for function and depend on each other. The existence of the secondary binding sites makes it possible to reconcile findings of negative cooperativity [48] with reports on two substrates bound to an aaRS dimer [33, 34].

Previous studies have attempted to detect binding events of aaRS substrates using kinetic assays in order to define a potential catalytic cycle in which aaRS charges its cognate tRNA. They proposed a so‐called flip‐flop mechanism, where each aaRS monomer performs the aminoacylation and transfer reaction in an alternating manner [34, 49, 50]. This mode of action has not only been suggested for aaRS; other enzymes have also been reported to function according to the alternating principle [51]. However, ultimate proof of the use of this mechanism by aaRS has remained elusive. By reviving their mechanistic suggestion with the addition of our recent findings, we present a potential catalytic cycle for the PylRS dimer (Figure 5). First, ncAA binds to one of the catalytic binding sites, which destabilizes the binding of a further ligand to the second active site and allows the positioning of a second ncAA molecule at the secondary binding site of the other dimer subunit. Our in vitro experiments have demonstrated that these binding events can happen in the absence of tRNA. In the next step, tRNA can be charged and released. The second ncAA can be relocated from its “waiting position” to the other catalytic binding site, which increases aaRS efficiency upon the pre‐positioning of substrates. In the in vitro experiments, neither ATP nor tRNA was present. However, we have strong evidence from our in‐cell experiments that the secondary binding sites are occupied under physiological conditions (i.e., inside living *E. coli* cells), hinting at a role in the catalytic cycle.

**FIGURE 5:**
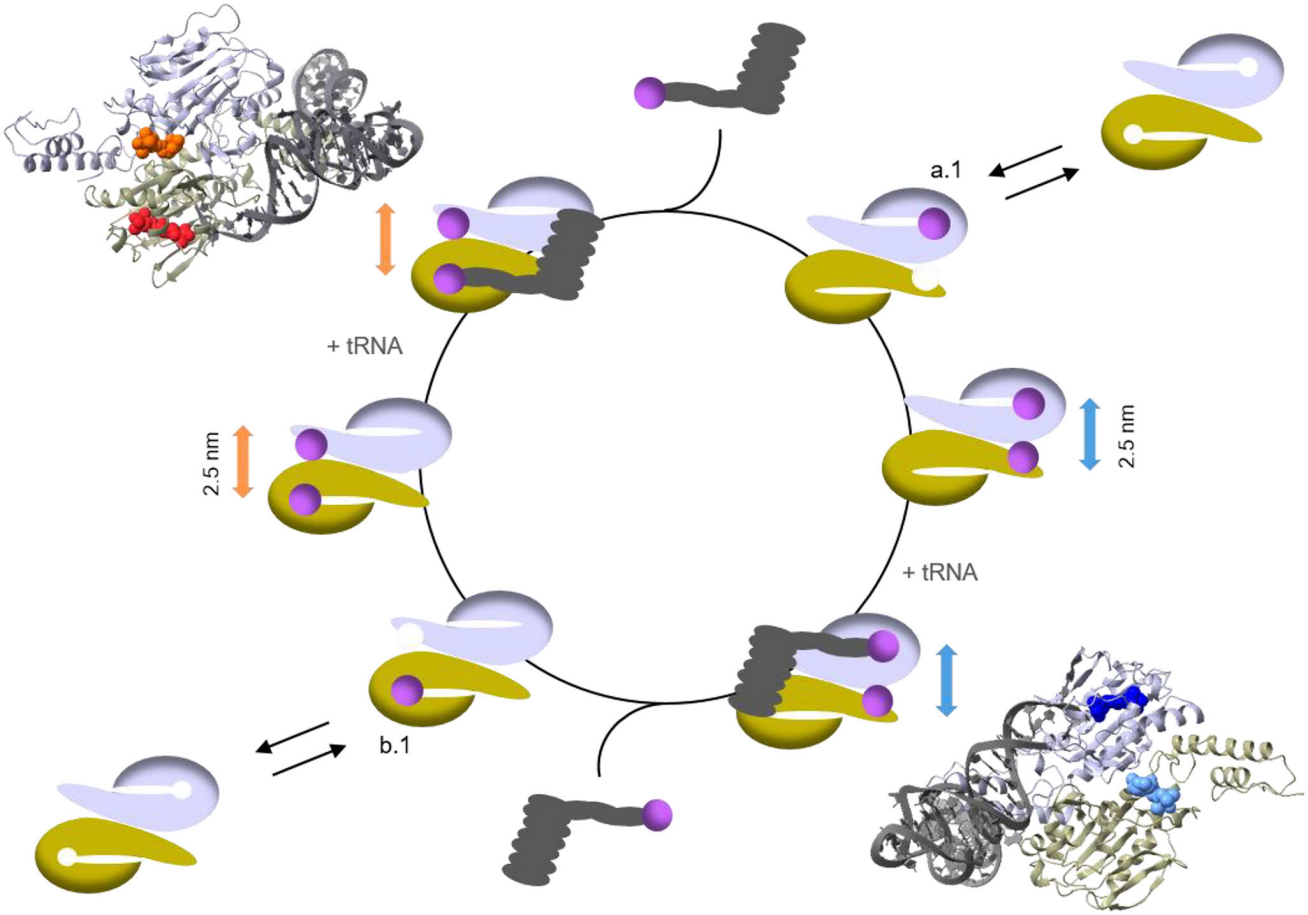
Proposed model for the catalytic cycle of the charging tRNA^Pyl^ by the PylRS dimer pair. In the model, ncAA (purple) is binding to the catalytic binding site a.1 *or* b.1 (start). Once ncAA is bound, the secondary binding site on the other PylRS monomer becomes accessible and is occupied. Then, tRNA^Pyl^ (blue) binds and is charged with ncAA at the catalytic binding site. Upon release of the charged tRNA^Pyl^, the ncAA at the secondary site is relocated to the catalytic binding site of the other PylRS monomer. Insets: Modeled structure of the PylRS dimer with two ncAAs and tRNA bound to it. One ncAA is bound to the catalytic binding site, and the other molecule is bound at the secondary binding site of the other PylRS monomer. The interaction of the ncAA with the secondary binding site does not influence the binding and charging of tRNA.

Besides the flip‐flop mechanism, other possibilities have been suggested in the literature [6, 52, 53]. Class I aaRS, which mainly exist as monomers [5], are especially thought to exhibit other tRNA charging reactions. However, class II aaRS obligatorily occur as dimers or even multimers, which argues in favor of a commonly shared mechanism. We can therefore assume that our proposed catalytic cycle is not an isolated phenomenon; it might rather occur in other aaRS systems as well.

## Conflicts of Interest

The authors declare no conflicts of interest.

## Supporting information




**Supporting File**: anie71776‐sup‐0001‐SuppMat.docx.

## Data Availability

The data that support the findings of this study are openly available in KonDATA at https://doi.org/10.48606/102.
