# Supplementary material for: Unraveling Synthetase's Mode of Action: The Pyrrolysyl‐tRNA Synthetase Dimer Uses Secondary Binding Sites in the Cell
Source: Angew Chem Int Ed Engl. 2026 Mar 24;65(18):e14065. doi: 10.1002/anie.202514065 (PMC13110774; doi:10.1002/anie.202514065)
Supplement: Supplementary file 1 — Supporting File: anie71776‐sup‐0001‐SuppMat.docx. [file ANIE-65-e14065-s001.docx]

Supporting Information
©Wiley-VCH 2021
69451 Weinheim, Germany

Unraveling Synthetase’s Mode of Action: The Pyrrolysyl-tRNA Synthetase Uses Secondary Binding Sites in the Cell

Jessica Dröden, Christoph Globisch, Eliane Landwehr, Theresa S. Braun, Daniel Summerer, Christine Peter, and Malte Drescher*

**Abstract:** Aminoacyl-tRNA synthetases mediate the activation and transfer of amino acids to their cognate tRNA, which constitutes one of the initial events in protein biosynthesis. Even though different mechanisms of action have been proposed for catalysis of these enzymes, their entire catalytic cycle remains elusive. Here, we used electron paramagnetic resonance spectroscopy *in vitro* and in cells in combination with molecular dynamics simulations to study the role of amino acid interactions in the catalytic cycle of pyrrolysyl-tRNA synthetases (PylRS), a widely used tool for genetic code expansion. Experiments using the paramagnetic non-canonical amino acid SLK-1 revealed the presence and occupation of secondary amino acid binding sites in PylRS located at the intermonomer interface, distant from the catalytic binding site. Based on our results, we propose a model that assumes an alternating mode of action of the two PylRS monomers for the catalytic cycle of PylRS.

DOI: 10.1002/anie.2021XXXXX

**Table of Contents**

[Experimental Procedures 3](#_Toc201868299)

[Expression and purification of C-terminal fragment of PylRS 3](#_Toc201868300)

[Cloning, expression, and purification of the C-terminal fragment of PylRS-SL1 3](#_Toc201868301)

[*In vitro* SLK-1 experiments 3](#_Toc201868302)

[In-cell SLK-1 experiments 4](#_Toc201868303)

[Continuous wave EPR spectroscopy 4](#_Toc201868304)

[Double electron-electron resonance (DEER) experiments 4](#_Toc201868305)

[Functionality assay of the tRNA^Pyl^/PylRS-SL1 pair 4](#_Toc201868306)

[Gel electrophoresis 4](#_Toc201868307)

[Estimation of PylRS-SL1 dimer occupation with SLK-1 5](#_Toc201868308)

[Molecular docking 6](#_Toc201868309)

[MD simulations of predocked ligands 6](#_Toc201868310)

[MD simulations with free ligands in solution 7](#_Toc201868311)

[Analysis and visualization of docking results and MD simulations 7](#_Toc201868312)

[Supplementary Figures 8](#_Toc201868313)

[**Figure S1.** cdPylRS-SL1 forms dimers *in vitro*. 8](#_Toc201868314)

[**Figure S2.** *In vitro* EPR data of cdPylRS-SL1 dimer after incubation with SLK-1 at varying dimer:SLK-1 ratios with or without washing procedure. 9](#_Toc201868315)

[**Figure S3.** Simulations of the *in vitro* EPR spectrum of cdPylRS-SL1 dimer in presence of SLK‑1. 10](#_Toc201868316)

[**Figure S4.** Positioning of SLK-1 within the hydrophobic catalytic binding site of cdPylRS-SL1. 10](#_Toc201868317)

[**Figure S5.** SLK-1 displacement within the catalytic binding sites. 11](#_Toc201868318)

[**Figure S6.** SLK-1 displacement within the secondary binding sites in MD simulations after initial docking into secondary binding sites of the X-ray derived models L1 and L2. 12](#_Toc201868319)

[**Figure S7.** *In vitro* DEER measurements of cdPylRS-SL1 in presence of SLK‑1. 13](#_Toc201868320)

[**Figure S8.** Echo-detected field sweep of cdPylRS in presence of SLK-1. 13](#_Toc201868321)

[**Figure S9.** Comparison of simulated vs. experimental *in vitro* distances. 14](#_Toc201868322)

[**Figure S10.** Contact analysis for simulations with 8 excess ligands in solution and predocked ligands in both catalytic sites (for protein models L1 (subfigure A) and L2 (subfigure B)). 15](#_Toc201868323)

[**Figure S11.** Contact analysis for simulations with 10 excess ligands in solution and empty catalytic sites (for protein models L1 (subfigure A) and L2 (subfigure B)). 17](#_Toc201868324)

[**Figure S12.** Functionality assay of PylRS-SL1 and its cognate tRNA^Pyl^. 18](#_Toc201868325)

[**Figure S13.** In-cell EPR spectra. 19](#_Toc201868326)

[**Figure S14.** SLK-1 does not bind nonspecifically to cellular components. 19](#_Toc201868327)

[**Figure S15.** In-cell echo-detected field sweep of cdPylRS in presence of SLK-1. 20](#_Toc201868328)

[**Figure S16.** In-cell DEER measurement of cdPylRS-SL1 in presence of SLK‑1. 20](#_Toc201868329)

[**Figure S17.** In-cell DEER measurement of PylRS-SL1 in presence of SLK‑1. 21](#_Toc201868330)

[**Figure S18.** DEER raw data of in-cell DEER measurements in presence of SLK‑1. 21](#_Toc201868331)

**Figure S19.** Investigating orientation selectivity

[**Figure S20.** Purification of cdPylRS. 22](#_Toc201868332)

[**Figure S21.** Sequence alignment of cdPylRS (gray, Seq 1) and cdPylRS-SL1 (black, Seq 2). 24](#_Toc201868333)

[**Figure S22.** Purification of cdPylRS-SL1. 24](#_Toc201868334)

[References 25](#_Toc201868335)

Experimental Procedures

Expression and purification of C-terminal fragment of PylRS

Plasmids containing the C-terminal fragment of *M. mazei* PylRS (pEVOL_PylRS_AF_188-454-His_6_), hereafter named cdPylRS, were kindly provided by the D. Summerer group (Technical University Dortmund, Dortmund, Germany)^[1]^ and transformed into *E. coli* BL21 Gold (DE3) cells (Agilent). Expression and purification was performed as described previously elsewhere.^[1]^ Briefly, 1 L lysogeny broth (LB) medium (Carl Roth) supplemented with 34 µg/mL chloramphenicol (Carl Roth) was inoculated with 20 mL of an overnight culture (dilution 1:50) and incubated at 37 °C and 160 rpm. Induction was performed at OD_600_ = 0.4 – 0.6 with 0.02 % L-arabinose (Carl Roth). After expression for 4 h at 37 °C and 160 rpm, the cells were harvested by centrifugation. The pellet was resuspended in 20 mL B-PER lysis reagent (Thermo Fisher) supplemented with EDTA-free cOmplete protease inhibitor cocktail (Roche) and shaking was performed at room temperature for 20 min. Afterward, the mixture was pelleted by centrifugation at 4 °C and maximum speed for 30 min. The supernatant was recovered and 400 µL 500 mM imidazole and 500 µL Ni-NTA agarose (Qiagen) were added. The mixture was shaken at room temperature for 30 min and then transferred to a gravity flow column. The resin was washed four times with 5 mL PBS, two times with 2 mL wash buffer 1 (50 mM NaH_2_PO_4_, 300 mM NaCl, 20 mM imidazole, pH 8.0), and two times with 2 mL wash buffer 2 (50 mM NaH_2_PO_4_, 300 mM NaCl, 50 mM imidazole, pH 8.0) using gravity flow. Elution was performed in three steps using 500 µL elution buffer (50 mM NaH_2_PO_4_, 300 mM NaCl, 500 mM imidazole, pH 8.0). The success of purification was verified using SDS-PAGE and ProBlue Safe Stain (Giotto Biotech) (Figure S19A). Afterward, elution fractions were further purified by size exclusion chromatography using a Superdex 200 column equilibrated in 10 mM HEPES, 300 mM NaCl, 5 mM MgCl_2_, pH 7.4. Fractions containing cdPylRS were chosen according to SDS-PAGE (Figure S19B), combined and concentrated using Vivaspin 20 centrifugal filters (10 MWCO, Sartorius). h

Cloning, expression, and purification of the C-terminal fragment of PylRS-SL1

For the construction of the SLK-1 binding fragment PylRS_AF_I413L, hereafter named cdPylRS-SL1 following Schmidt et al.,^[2]^ quick-change mutagenesis was performed using pPylRS_AF_188-454-His_6_ and primers oJD64 (CTG GAC CGT GAG TGG GGT CTG GAC AAA CCG TGG ATC G) and oJD65 (GAT CCA CGG TTT GTC CAG ACC CCA CTC ACG GTC CAG C). As revealed by Sanger sequencing, the resulting plasmid contained the I to L mutation at the desired position, but lacked the His_6_-tag (Figure S20). Transformation and expression of this plasmid was performed as described above. Due to the missing His_6_-tag, the purification protocol was changed to purify the cdPylRS-SL1 construct. The pellet was resuspended in 20 mL anion-exchange buffer (50 mM NaH_2_PO_4_, pH 8.0) supplemented with cOmplete protease inhibitor cocktail (Roche) and 1 mM 1,4-Dithiothreitol (DTT) and incubated for 10 min on ice. Lysis was performed with a Q700 SONICATOR (QSONICA) equipped with a 6.4 mm microtip probe using an amplitude of six with the pulse on for 1 s and the pulse off for 1 s for 2 min. Afterward, the mixture was centrifuged at 4 °C and maximum speed for 30 min. The supernatant was recovered, filtered and loaded onto a HiTrap Q HP column (2 x 5 mL, GE Healthcare) equilibrated in anion-exchange buffer. Elution was performed using a step-wise gradient (40 mL, 0-100 %) of anion-exchange elution buffer (50 mM NaH_2_PO_4_, 1 M NaCl, pH 8.0). Collected fractions were analyzed using SDS-PAGE and ProBlue Safe Stain (Figure S21A). Fractions containing cdPylRS_I413L_cd were combined and further purified by size exclusion chromatography using a Superdex 200 column equilibrated in 10 mM HEPES, 300 mM NaCl, 5 mM MgCl_2_, pH 7.4. Fractions containing cdPylRS-SL1 (Figure S21B) were combined and concentrated using Vivaspin 20 centrifugal filters, 10 MWCO (Sartorius).

*In vitro* SLK-1 experiments

For the *in vitro* experiments, the purified catalytic fragments were thawed and their concentration was estimated spectrophotometrically by UV absorption at 280 nm (BioPhotometer D30, Eppendorf) using an extinction coefficient ε = 21680 M^-1^cm^-1^. To achieve the binding of SLK-1, protein sample was mixed with SLK-1 (synthesized by A. Fedoseev and R. Steimbach in our group, as previously described elsewhere^[2]^) and incubated for 1 h at 37 °C and 300 rpm (Thermomixer C, Eppendorf) resulting in a dimer concentration of 33 µM and 15 equivalents (500 µM) of SLK-1. Afterward, the mixtures were transferred to Amicon Ultra 0.5 mL centrifugal filters, 10 MWCO (Merck Millipore) and washed eight times by centrifugation at 4 °C and 14,000 g for 6 min with 200 µL 10 mM HEPES, 300 mM NaCl, 5 mM MgCl_2_, pH 7.4. The sample was recovered, re-adjusted to the initial dimer concentration, filled into glass capillaries (HIRSCHMANN^®^ ringcaps^®^, inner diameter 1.02 mm) and sealed with Hemato-Seal™ capillary tube sealant (Fischer-brand™). Subsequently, EPR spectra were recorded. For double electron-electron resonance (DEER) experiments, 48 µL of sample were mixed with 12 µL d_8_-glycerol (20 % *v/v*, Sigma), transferred into 3 mm outer-diameter quartz tubes (Fused quartz tubing, Technical Glass Products) and flash frozen in liquid nitrogen.

In-cell SLK-1 experiments

In-cell experiments were either performed in *E. coli* BL21 Gold cells containing cdPylRS-SL1 plasmid, *E. coli* GH371 cells containing tRNA^Pyl^/PylRS-SL1 plasmid (pEVOL-based plasmid encoding full-length synthetase and the corresponding tRNA^[2]^) or *E. coli* GH371 cells alone. For growth and expression, 20 mL LB medium supplemented with the respective antibiotic (chloramphenicol for plasmid-containing cells, non for GH371 cells only) were inoculated with 1 % of an overnight culture and incubated at 37 °C and 180 rpm. Induction was performed at OD_600_ = 0.6 – 0.8 with 0.2 % L-arabinose and 1 mM SLK-1 (synthesized by A. Fedoseev and R. Steimbach in our group as previously described elsewhere^[2]^) was added. After expression for 5 h at 37 °C and 180 rpm, cells were harvested by centrifugation. Cells were subsequently washed three times with pre-cooled LB medium containing 20 % glycerol (*v/v*) by centrifugation at 4 °C and 4000 g for 6 min. Afterward, cells were washed once with 1 mL pre-cooled LB medium and the pellet was resuspended in the remaining medium after discarding the supernatant. For EPR experiments, 25 µL of the cell solution were filled in glass capillaries, and the spectra were recorded directly. For DEER experiments, 48 µL of the cell solution were mixed with 12 µL d_8_-glycerol (20 % *v/v*) and flash frozen in liquid nitrogen.

Continuous wave EPR spectroscopy

EPR spectra were recorded at room temperature and at X-band microwave frequency (9.645 GHz) with the benchtop spectrometer EMXnano (Bruker), equipped with a cylindrical cavity mode TM_110_. The center field was set to 3430 G using a sweep width of 180 G and a sweep time of 30 s. The modulation amplitude was set to 1.5 G, the microwave attenuation to 15 dB, and the modulation frequency to 100 kHz. Five to ten scans were averaged (for *in vitro* experiment, 60 scans were averaged). The recorded spectra were processed with MatLab R2023a (The MathWorks, Inc.) and the toolbox EasySpin 5.2.35.^[3]^

Double electron-electron resonance (DEER) experiments

DEER experiments were performed at Q-band frequency (34 GHz) and 50 K with an Elexsys E580 spectrometer (Bruker), equipped with an arbitrary waveform generator (AWG) unit (Bruker) and a 150 W traveling tube (TWT) amplifier (Applied Systems Engineering) in a commercial Q-band resonator (ER5106QT2, Bruker). The temperature was controlled by a cryogen-free helium recirculation system with a ColdEdge cryocooler (CE-FLEX-4K_0100, Bruker), a F-70H helium compressor (SHI cryogenics), and a Mercury ITC temperature controller (Oxford Instruments). Pulsed experiments were performed with the standard four-pulse DEER sequence (π/2_obs_ – τ_1_ – π_obs_ – (τ_1_ + t) – π_pump_ – (τ_2_ - t) – π_obs_ – τ_2_ – echo)^[4]^ with rectangular pump and observer pulses. The pump frequency was set to 34 GHz. A frequency offset of 70 MHz (33.93 GHz) was chosen for the observer pulse. The length of the pump pulse was set to 16 or 18 ns and the lengths of the observer π pulses were set to 24-28 ns. Nuclear modulation averaging was done by increasing τ_1_ in eight steps of 16 ns each. Artifacts caused by interfering echoes were removed with an 8‑step on pump {(x) x [xp] x} phase cycling.^[5]^ For *in vitro* experiments, scans were accumulated overnight for 15-20 hours. For in-cell experiments, scans were accumulated for up to 48 hours due to low signal-to-noise ratios. The processing of the raw DEER data was performed with MatLab R2023a (The MathWorks, Inc.) and the toolbox EasySpin 5.2.35^[3]^. For unbiased analysis, the Comparative DEER Analyzer (CDA) within DeerAnalysis 2022^[6]^ was used.

Functionality assay of the tRNA^Pyl^/PylRS-SL1 pair

The incorporation of SLK-1 into the protein α-synuclein was performed as previously described elsewhere.^[2,7]^ In brief, *E. coli* BL21 Gold (DE3) cells were transformed with pEVOL-based tRNA^Pyl^/PylRS-SL1 plasmid^[2]^ and pT7-asyn-S9TAG plasmid containing an amber stop codon at position 9 in the amino acid sequence. 20 mL of LB medium supplemented with 50 mg/mL carbenicillin and 34 µg/mL chloramphenicol was inoculated with 1 % (v/v) of an overnight culture. Expression was induced at OD_600_ = 0.7 with 1 mM IPTG and 0.2 % (w/v) L-arabinose in the presence of 3 mM SLK-1. Expression was stopped via centrifugation after 6 h. The cell pellet was resuspended in 500 µL B-PER™ (Thermo Fisher Scientific) containing 1 mM phenylmethylsulfonyl fluoride (PMSF, Carl Roth) and incubated for 10 min on ice. The cell debris was removed via centrifugation. The supernatant was treated with 30 % (w/v) ammonium sulphate (Carl Roth) to precipitate the proteins. Precipitated proteins were harvested via centrifugation and further analyzed via SDS-PAGE analysis.

Gel electrophoresis

Each sample was mixed with 6*x* Laemmli sample buffer to a final volume of 24 µL. The samples were boiled for 5 min at 95 °C for denaturation. Afterward, 10 µL of each sample were loaded on 15 % SDS gels including a reference broad range protein marker (Precision Plus Protein™ Standard, Bio-Rad Laboratories, Inc.). SDS gels were run at 130 V and then, gels were stained with ProBlue Safe Stain (Giotto Biotech). The gels were subsequently transferred into milliQ water and incubated overnight to remove background staining. For documentation, the ChemiDoc MP Imaging System (Bio-Rad Laboratories, Inc.) was used.

For semi-native gel electrophoresis, samples were mixed with 6*x* Laemmli sample buffer without SDS and no boiling was performed to avoid denaturation of the native protein structure.

Estimation of PylRS-SL1 dimer occupation with SLK-1

To estimate the concentrations of unbound SLK-1, singly occupied cdPylRS-SL1 dimer and doubly occupied cdPylRS-SL1 dimer, information from both CW EPR and DEER experiment is necessary.

From the EPR spectrum of the *in vitro* sample (Figure 1B), the total spin concentration was determined to be 14 µM using the built-in EMXnano reference-free spin counting module (Xenon software, Bruker). The line shape of the EPR spectrum indicates that the sample contains bound and unbound SLK-1. We simulated the spectrum taking both components into account. Simulations were performed in MatLab R2023a (The MathWorks, Inc.) using the toolbox EasySpin 5.2.35.^[3]^ For the simulation, an isotropic rotation of the nitroxide (S = ½) with the following g-tensor values was utilized: g = [2.00906 2.00687 2.00300]. The g-tensor was chosen based on the literature.^[8]^ A superposition of two components was used to simulate the spectrum using the “chili” function of the EasySpin^[3]^ toolbox. The values of the 14N hyperfine coupling tensor were Axx = Ayy = 13 MHz and Azz = 110 MHz. A Gaussian component with a linewidth of 0.12 mT was used. The obtained rotational correlation times τ_r_ were 0.06 ns for the fast component, and 7.9 ns for the slow component. The superpositions of the experimental spectrum and of the simulation are shown in Supplementary Fig. S2. We determined the fraction of the fast component (unbound SLK-1) to be 8 %, whereas the slow component (bound SLK‑1) corresponded to 92 %.

In DEER experiments, the modulation depth gives information about the fraction of coupled spins. The modulation depth λ can be described as:^[9]^

| $\lambda=1-[f_{2}\left( 1-p_{B} \right)+f_{1}]$ | (1) |
| --- | --- |

with

| $f_{1}=1-f_{2}$ | (2) |
| --- | --- |

where $f_{1}$ is the fraction of single spin species (here: unbound SLK-1 or singly occupied PylRS dimer) and $f_{2}$ is the fraction of species containing coupled spins (here: doubly occupied PylRS dimer). $p_{B}$ is the probability of the DEER pump pulse to excite spins B. $p_{B}$ equals the maximum achievable modulation depth $\lambda_{max}$ for a sample containing coupled spins only (i.e., $f_{1}=0$ and $f_{2}=1$). $\lambda_{max}$ was determined experimentally to be 0.31 for the same setup and parameters used in this study.^[10]^

Using (1) and (2) with the average modulation depth from three replicates (Fig. 3A, Fig. S6) of the *in vitro* cdPylRS-SL1 sample ($\lambda=0.09$), it is calculated that $f_{1}=0.71$ and $f_{2}=0.29$.While the stability of SLK-1 against reduction is limited in E.coli, it is stable in vitro. Therefore, we assume that SLK-1 remains paramagnetic to 100% in our in vitro experiments. $f_{1}$ and $f_{2}$ can be expressed as:

| $f_{1}=\frac{c_{0}+c_{1}}{c_{tot}}$ | (3) |
| --- | --- |
|  |  |
| $f_{2}=\frac{c_{2}}{c_{tot}}$ | (4) |

with $c_{0}$, $c_{1}$, $c_{2}$ being the concentrations of unbound SLK-1, singly occupied cdPylRS-SL1 dimer, and doubly occupied cdPylRS-SL1 dimer, respectively, and $c_{tot}=c_{0}+c_{1}+c_{2}$. Similarly, the concentrations of spins belonging to unbound SLK-1, singly and doubly occupied cdPylRS-SL1 dimer are described with:

| $c_{tot}^{*}=c_{0}^{*}+c_{1}^{*}+c_{2}^{*}$ | (5) |
| --- | --- |

For unbound SLK-1 and singly occupied cdPylRS-SL1 dimer, $c_{0}{=c}_{0}^{*}$ and $c_{1}{=c}_{1}^{*}$. For doubly occupied dimer, $c_{2}{=\frac{1}{2}c}_{2}^{*}$. Thus, $f_{1}$ and $f_{2}$ can also be expressed as:

| $f_{1}=\frac{c_{0}^{*}+c_{1}^{*}}{c_{0}^{*}+c_{1}^{*}+\frac{1}{2}c_{2}^{*}}$ | (6) |
| --- | --- |
|  |  |
| $f_{2}=\frac{\frac{1}{2}c_{2}^{*}}{c_{0}^{*}+c_{1}^{*}+\frac{1}{2}c_{2}^{*}}$ | (7) |

From (5) and (7), the concentration of coupled spins is calculated to be 6 µM with:

| $c_{2}^{*}=\frac{2f_{2}c_{tot}^{*}}{{1+f}_{2}}$ | (8) |
| --- | --- |

The concentration of spins bound to singly occupied dimer is calculated with (5) using the concentration of unbound SLK-1 determined from the EPR spectrum to be 7 µM.

Thus, the *in vitro* cdPylRS-SL1 sample is estimated to contain a total cdPylRS-SL1 dimer concentration of 33 µM, from which 7 µM is singly occupied (21 %), 3 µM is doubly occupied (9 %), and 23 µM is unoccupied (70 %).

Molecular docking

Docking was performed into two structural models of cdPylRS-SL1 based on the X-ray structure of *Methanosarcina mazei* PylRS(Y306A/Y384F) complexed with ZaeSeCys, pdb ID: 6AAP.^[11]^ The reason for two models originated in the reported alternative binding modes of the ligand and corresponding rotamers of residue ILE413 in the PDB structure. While introducing the mutation I413L, the reported side chain orientations of isoleucine were preserved in the leucine rotamers, which resulted in the models L1 and L2. Two different types of binding sites were explored. The catalytic binding sites a.1 and b.1 and the secondary binding sites a.2 and b.2 located at the interface between the monomers.

The docking of SLK-1 was performed with the GNINA 1.0 package.^[12,13]^ Models L1 and L2 were used for docking into the catalytic binding sites a.1 and b.1. To address the charged state of SLK‑1, AutoDock Tools 1.5.6^[14]^ was utilized to prepare the ligand input file. The box was defined based on the position of the native ligand in the pdb file with a default value of 4 Å added in each direction beyond the ligand size. The exhaustiveness was increased to 1000 and up to 30 models were calculated per docking run. For the simulations, the three best-scored ligand poses (CNN pose score) were selected for the protein models L1 and L2.

In an initial attempt, the protein models L1 and L2 have been used as the target protein structures for the docking into the secondary sites, a.2 and b.2, at the interface between the monomers. Later on, we repeated the docking into the secondary binding sites with representative structures of MD simulations. The combined six 500 ns long simulations of three SLK-1 ligand poses per protein model were clustered with an RMSD-based clustering^[15]^ and a cutoff value of 0.17 nm. The ten largest clusters were chosen for docking with GNINA. The selection of the poses for the subsequent MD simulation was based on the best score together with a distance range between the NO labels in the catalytic and secondary binding sites of 2.4 to 2.6 nm. For the final simulations the two clusters were selected with the SLK-1 orientation in the catalytic binding site closest to the initially docked position.

MD simulations of predocked ligands

The following systems were simulated. The first simulated three poses docked in the catalytic binding sites, a.1 and b.1, of protein models, L1 and L2, where both catalytic binding sites are occupied at the same time. An initial set of shorter simulations for one L1 and one L2 model with all four binding sites occupied were carried out as triplicates. For the final simulations exploring the secondary binding sites, only one catalytic and one secondary binding site were populated at the same time. One catalytic binding site, a.1 or b.1, together with one secondary site, a.2 or b.2, were carried out as quadruplicates. In total 28 MD simulations were performed.

All molecular dynamics simulations were obtained by GROMACS version 2021.4^[16,17]^. We used a combination of CgenFF^[18,19]^ for SLK‑1 and CHARMM36m^[20–22]^ for the protein and ATP together with the TIP3P water model. The force field parameters for the system have been obtained from the input generator tools in CHARMM-GUI^[20,23,24]^ using Ligand Reader & Modeler^[25]^ for the ligands and Solution Builder for the remaining parts. The initial positions of the ligands were obtained from docking experiments as described in the docking section.

The following simulation settings were applied. The simulation box was set to a dodecahedron with a minimum distance between the protein and the box of at least 2 nm. The system was solvated, and sodium and chloride ions were added to a concentration of 150 mmol/L. The leapfrog integrator^[26]^ was utilized with all bonds constrained by the LINCS algorithm^[27]^ to enable a time-step of 2 fs. We used a modified cutoff for short-ranged electrostatic and Lenard Jones interactions of 1.2 nm, with a switching function between 1.0 and 1.2 nm. Long-range Coulomb interactions were calculated using the particle mesh Ewald method (PME)^[28]^. Initially, all systems were energy minimized with the steepest-descent algorithm for 50000 steps. Two consecutive equilibration simulations followed in a canonical form (NVT - 100 ps), and later an isobaric-isothermal (NPT) ensemble where the solute atoms (protein and ligands - SLK-1 and ATP together with the bound magnesium ions) have been position restrained.

For the NPT simulations, two different equilibration lengths were applied. 100 ns for the systems with ligands solely bound to the catalytic binding site together with the initial set where all binding sites were occupied, and 1000 ps for the final systems where one ligand was bound to the secondary binding site.

The production simulations (NPT) were 500 ns long, despite the initial test setup with all binding sites being occupied with a length of 100 ns. Temperature and pressure were maintained at 298 K and 1 bar with the Nose-Hoover thermostat^[29,30]^ and the Parinello-Rahman barostat^[31]^ with a coupling time of 1 ps for the thermostat and 5 ps for barostat.

MD simulations with free ligands in solution

Motivated by the analysis of the simulations with predocked ligands where we observe dynamic binding of the ligands especially to the alternative binding site – unbinding, rebinding to the same or different alternative site and once even a motion towards an unoccupied enzymatic site –a further set of simulations was started with an excess of free ligands in solution in order to provide an independent view on the binding. Two scenarios were set up: one with predocked ligands in the enzymatic pockets and another one without aminoacid ligands in the enzymatic pocket (the cofactors ATP and Mg^2+^ were always present). The simulations with occupied enzymatic pockets focus on the binding to the secondary binding sites whereas the other setup is unbiased with respect to any prior docking. For both cases two protein starting structures corresponding to the two initial models L1 and L2 were used. The positions of predocked ligands also corresponded to the previous simulations. To these systems free ligands were added (8 in the predocked case or 10 in the case of unoccupied catalytic sites) – randomly placed in the simulation box. The simulation protocol was analogous to the previous simulations except for a massively extended production length of 2 µs per run.

Analysis and visualization of docking results and MD simulations

To describe the stability of the bound SLK-1 labels in their respective binding sites we calculated the root mean square deviation (RMSD) of the ligand with respect to its starting position in the production simulation after least square fitting of the binding site residues. This gives a relative displacement of the ligand from to its initial position in relation to the binding site.

To compare the distances between the bound ligands to those from the DEER experiments we calculated the distance between the oxygens of the nitroxide groups. We report two distributions: one (presented in solid lines) was computed over all frames of the simulation (irrespective of whether the ligands were actually bound or not). A second, distribution was computed (presented in dotted lines) where only those simulation timeframes were counted where both ligands are bound. The ligand was assigned as bound if the RMSD value with respect to its initial position was below 0.8 nm. This frame selection implies that the number of frames that were used to compute the distributions differ between the various distance pairs. We have accounted for that fact by rescaling (renormalizing) the distributions accordingly (by the fraction of selected frames with respect to all frames i.e. the total simulation length). This frame selection and scaling of the distance distributions reflects the experimental measurements where after a washing step only bound labels are present/detected.

Molecular graphics were produced with UCSF Chimera,^[32]^ developed by the Resource for Biocomputing, Visualization, and Informatics at the University of California, San Francisco, with support from NIH P41-GM103311, UCSF ChimeraX^[33,34]^ and visual molecular dynamics (VMD).^[35]^

Supplementary Figures


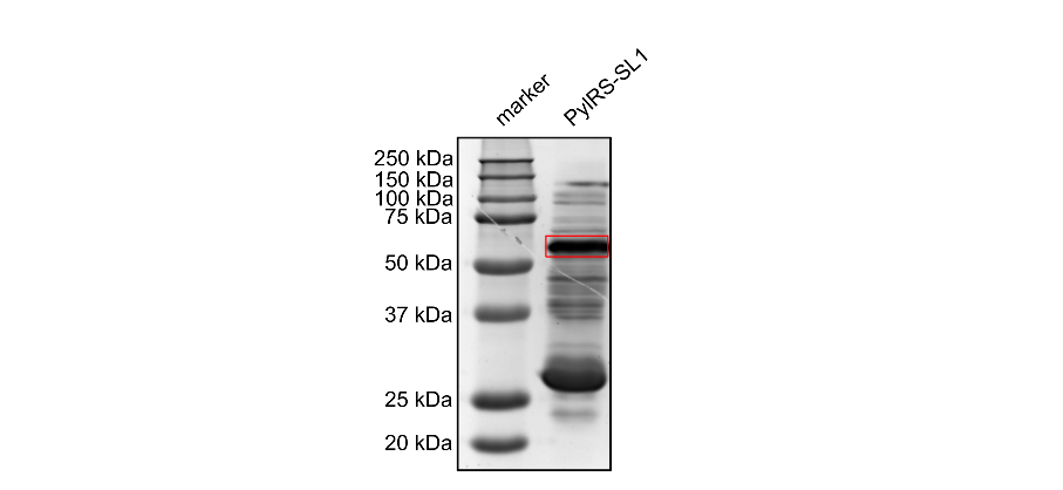


1. cdPylRS-SL1 forms dimers *in vitro*. Semi-native gel electrophoresis of purified cdPylRS-SL1. cdPylRS dimers of 62 kDa are unambiguously visible (red box).


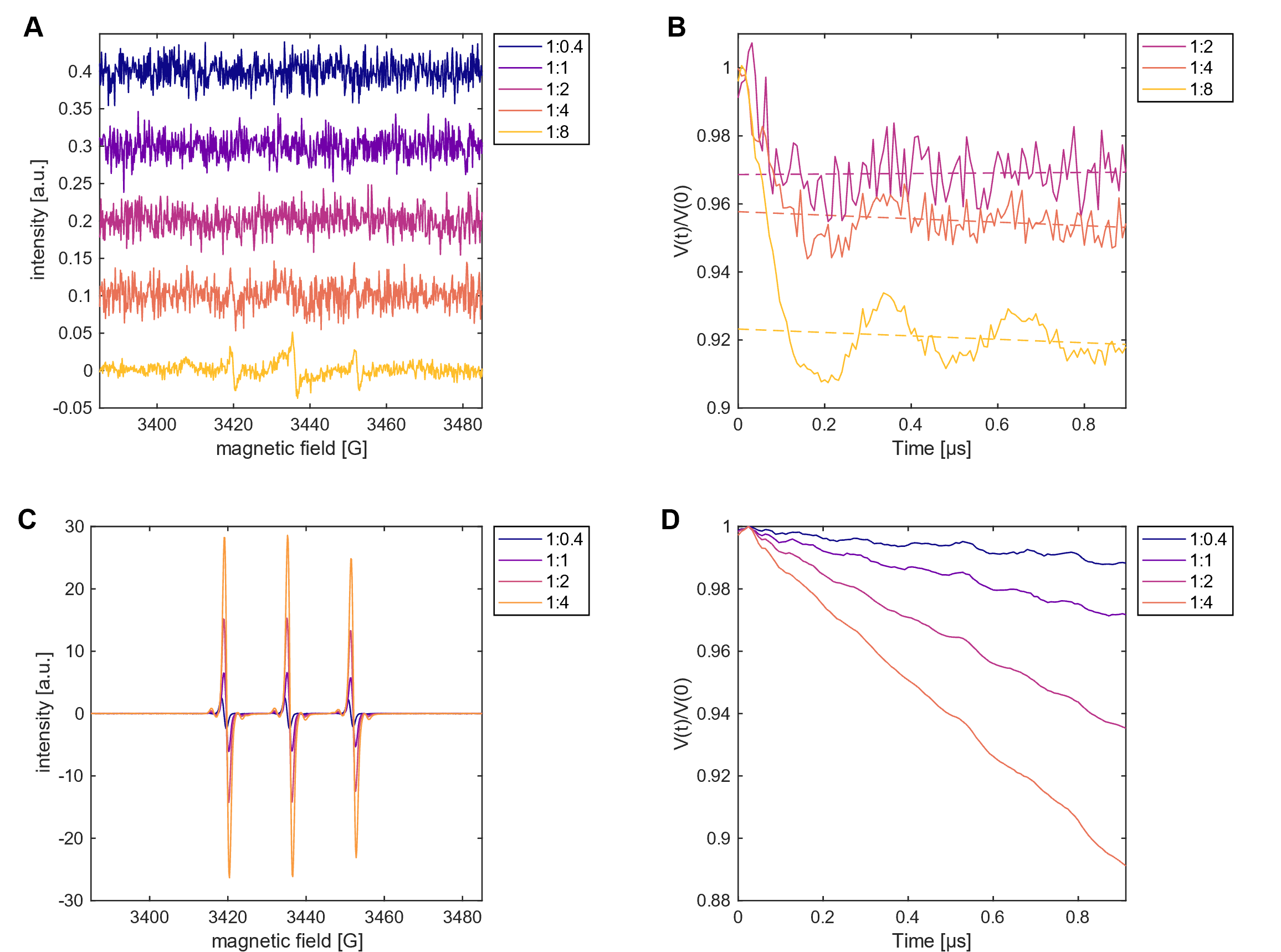


1. *In vitro* EPR data of cdPylRS-SL1 dimer after incubation with SLK-1 at varying dimer:SLK-1 ratios with or without washing procedure. Samples contain a cdPylRS-SL1 dimer concentration of 33 µM. (A) EPR spectra of washed samples. For dimer:SLK-1 ratios 1:0.4 to 1:4, 10 scans were averaged. For dimer:SLK-1 ratio 1:8, 100 scans were averaged. (B) DEER raw data and background (fitted in DeerAnalysis) for samples shown in (A) with dimer:SLK-1 ratios 1:2, 1:4 and 1:8. For dimer:SLK-1 ratios 1:0.4 and 1:1, the echo intensities were too low for DEER. (C) EPR spectra of unwashed samples (dimer:SLK-1 ratios of 1:0.4 to 1:4), indicating unbound SLK-1, only. (D) DEER raw data for samples shown in (C). Besides visible residual deuterium modulations all samples show a modulation depth of zero.


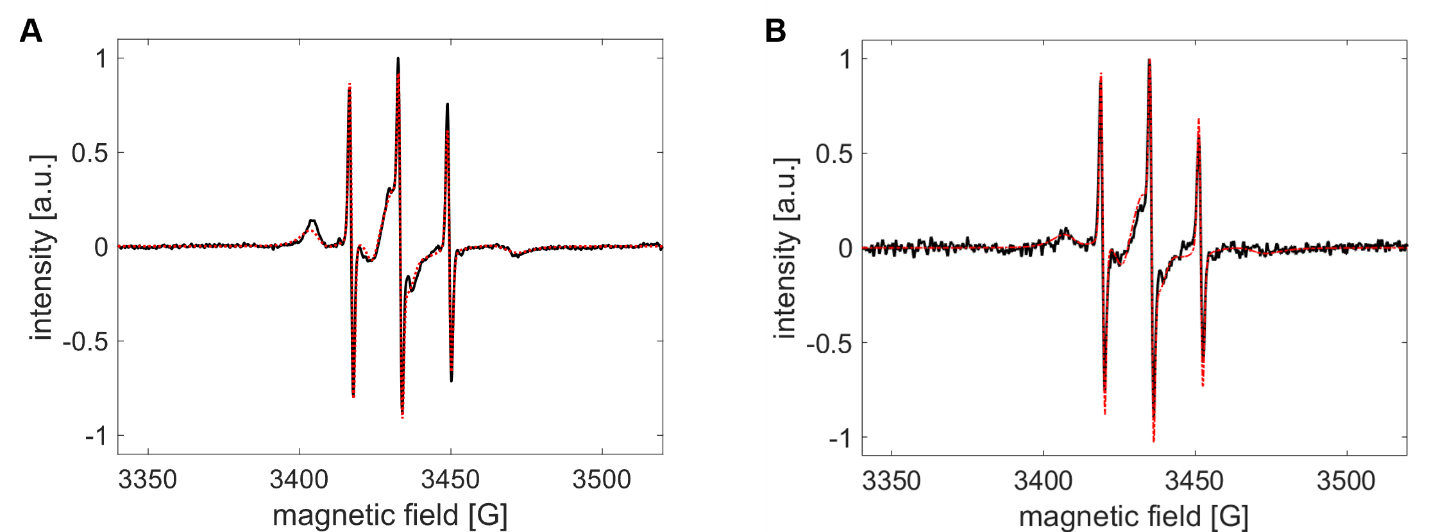


1. Simulations of the *in vitro* EPR spectrum of SLK‑1 in presence of cdPylRS-SL1 dimer. The experimental spectra (black) are superimposed with the simulated spectra (red). For simulation, two components with different rotational correlation times (τ_r,fast_ = 0.06 ns, and τ_r,slow_ = 7.9 ns for (A) or τ_r,slow_ = 6.3 ns for (B)) were used. The fraction of the fast component was determined to be approximately 8 %, whereas the slow component comprises approximately 92 %, respectively.

**
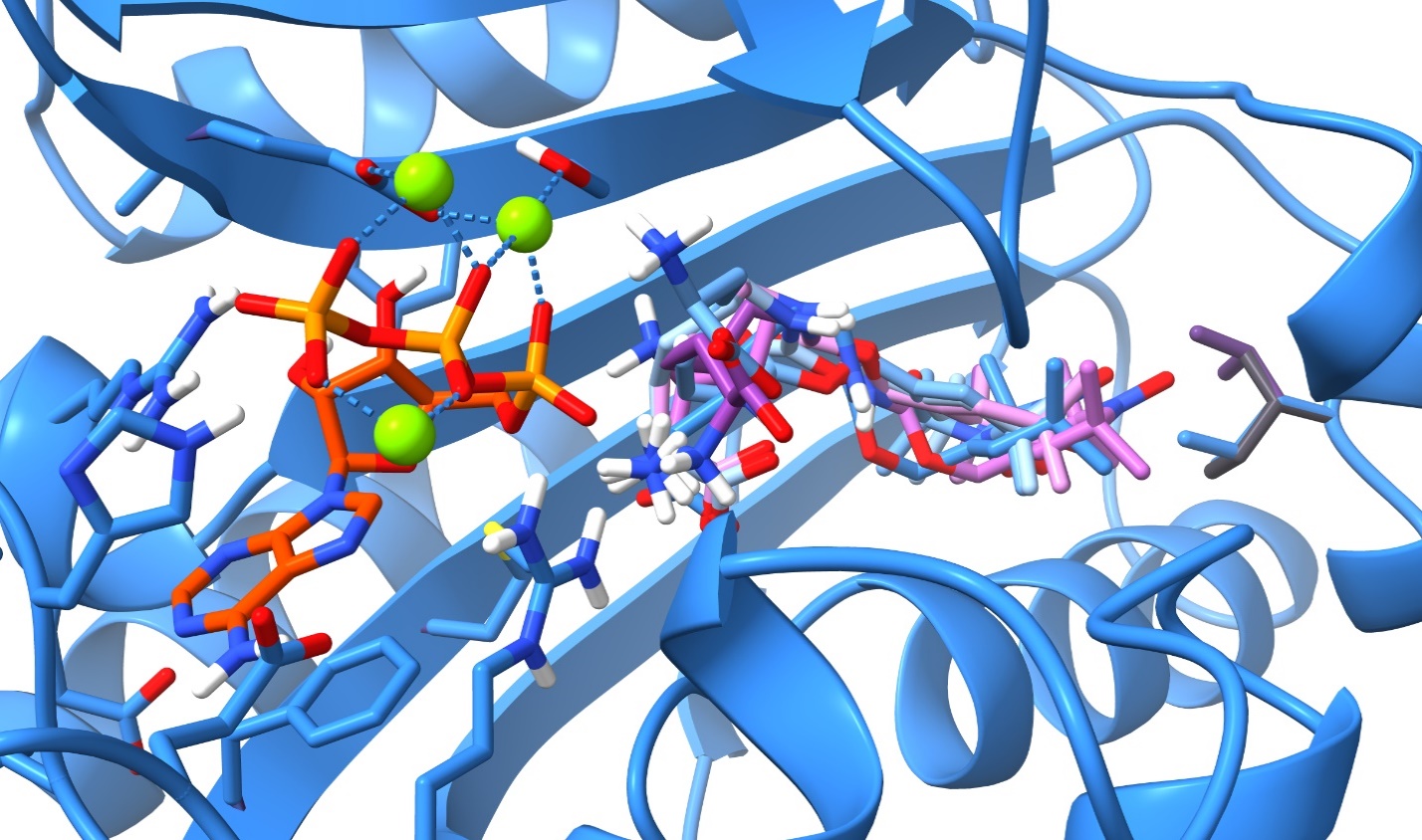
**

1. Positioning of SLK-1 within the hydrophobic catalytic binding site of cdPylRS-SL1. Two alternative models of the binding site have been used for docking according to leucine 413 rotamers, L1 (blue) and L2 (violet), following the branching of the wild type isoleucine 413 (gray). The different positions of L413 allow for slightly deeper binding modes of the SLK-1 label in model L2. Three best poses of SLK-1 docked into L1 (shades of blue) and L2 (shades of violet).


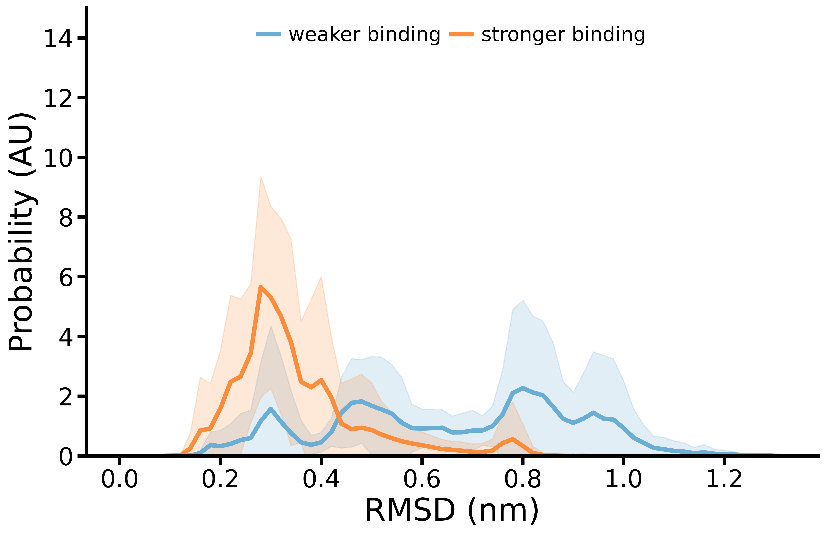


1. SLK-1 displacement within the catalytic binding sites. Distribution of root mean square displacement (RMSD) values of the SLK-1 ligand with respect to the initial docked position after least square fit of the binding site. Ligands were grouped according to higher and lower stability within a simulation, according to the observation that always one out of the two ligands bound to the (symmetry equivalent) catalytic sites was bound more weakly than the other.


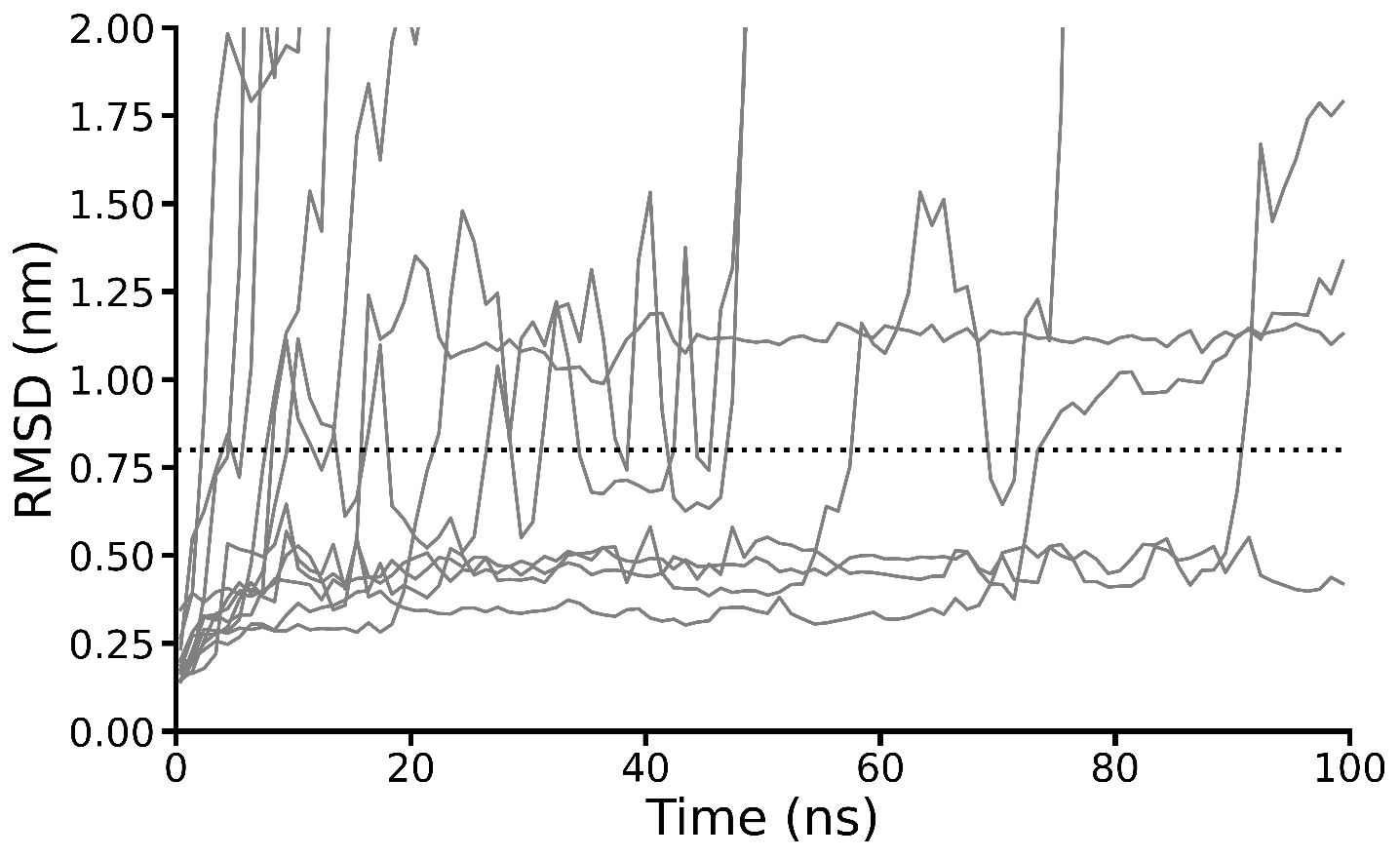


1. SLK-1 displacement within the secondary binding sites in MD simulations after initial docking into secondary binding sites of the X-ray derived models L1 and L2. Triplicate simulations for two models and two secondary binding sites result in 12 ligand time traces. Most ligands leave the secondary binding site after a few nanoseconds. Only one ligand remains bound on the timescale of 100 ns. This relatively unstable behavior was motivating the re-docking into equilibrated cluster representatives of only catalytic site occupied simulations.


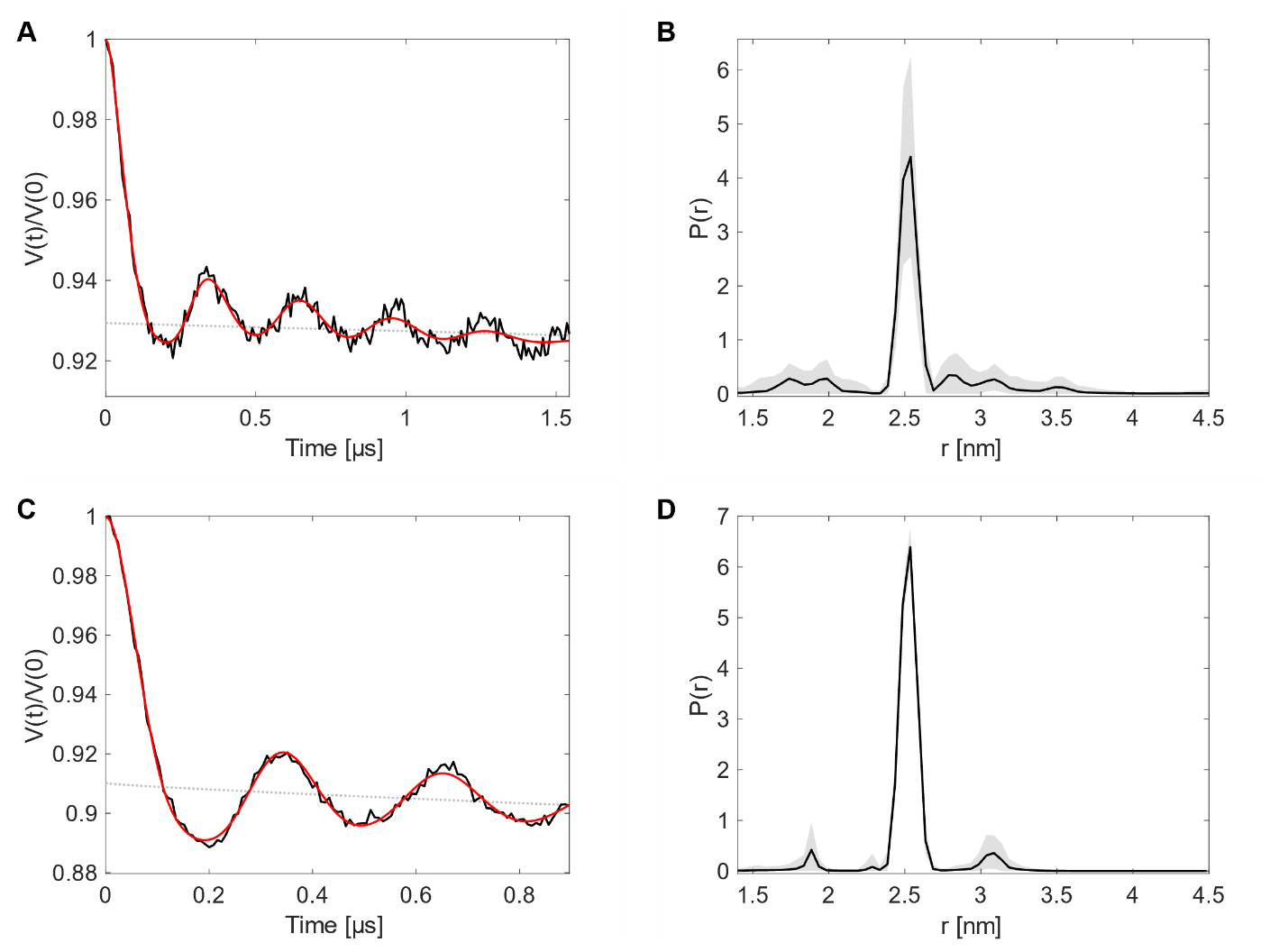


1. DEER measurements of SLK‑1 in presence of cdPylRS-SL1 *in vitro*. (A), (C) DEER raw data with background (gray) and fit (red). (B), (D) Consensus distance distributions including uncertainty (gray). Since the fit in (A) smears out the oscillations at longer times and orientation selectivity has been excluded (see Figure S19), this might suggest that the distance distribution might be even narrower than presented in (B).


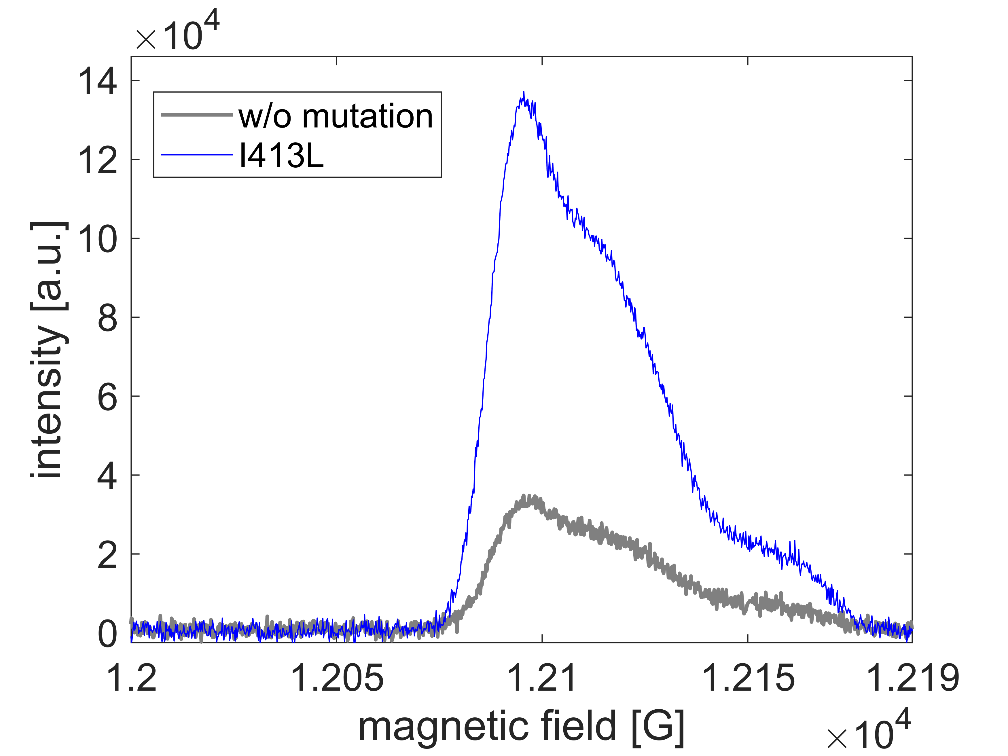


1. Echo-detected field sweep of SLK-1 in presence of cdPylRS-SL1 (blue) or cdPylRS (grey). Without the I413L mutation, the EPR nitroxide signal (grey) is attributed to unbound SLK-1.


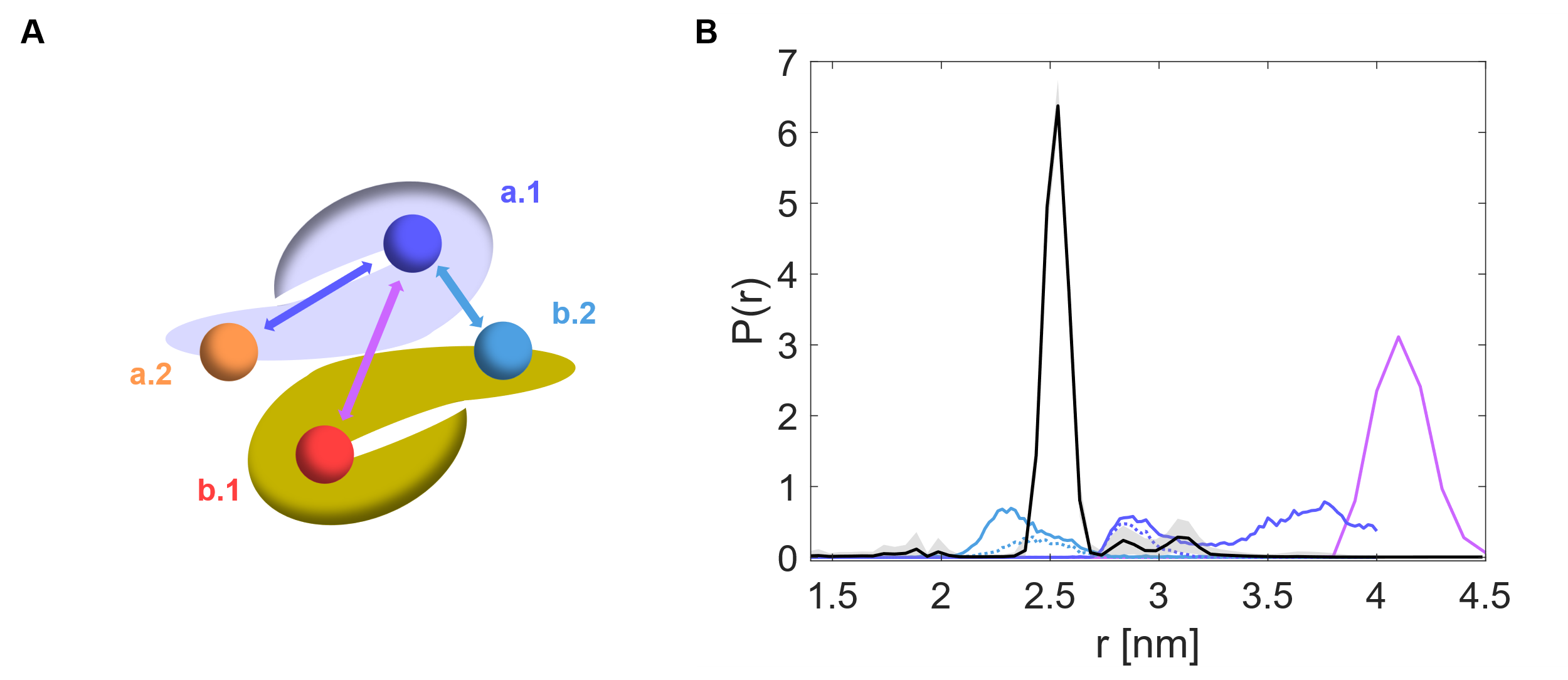


1. Comparison of simulated vs. experimental *in vitro* distances. (A) Schematic representation of cdPylRS-SL1 dimer and the different binding sites occupied with SLK-1. Three possible distances are indicated (complementary to the ones shown in Figure 3C). (B) Comparison of experimental consensus distance distribution (black) with simulated distance distributions (colored) of distances between SLK-1 pairs occupying three different combinations of binding sites (colored lines, the color code corresponds to the pairings indicated in (A)).The gray-shaded area indicates the uncertainty. The dotted distance distributions (yellow, brown) represent the probability distributions after accounting for the residencies of the ligands in the secondary binding sites in the different simulations. For details see the method section.


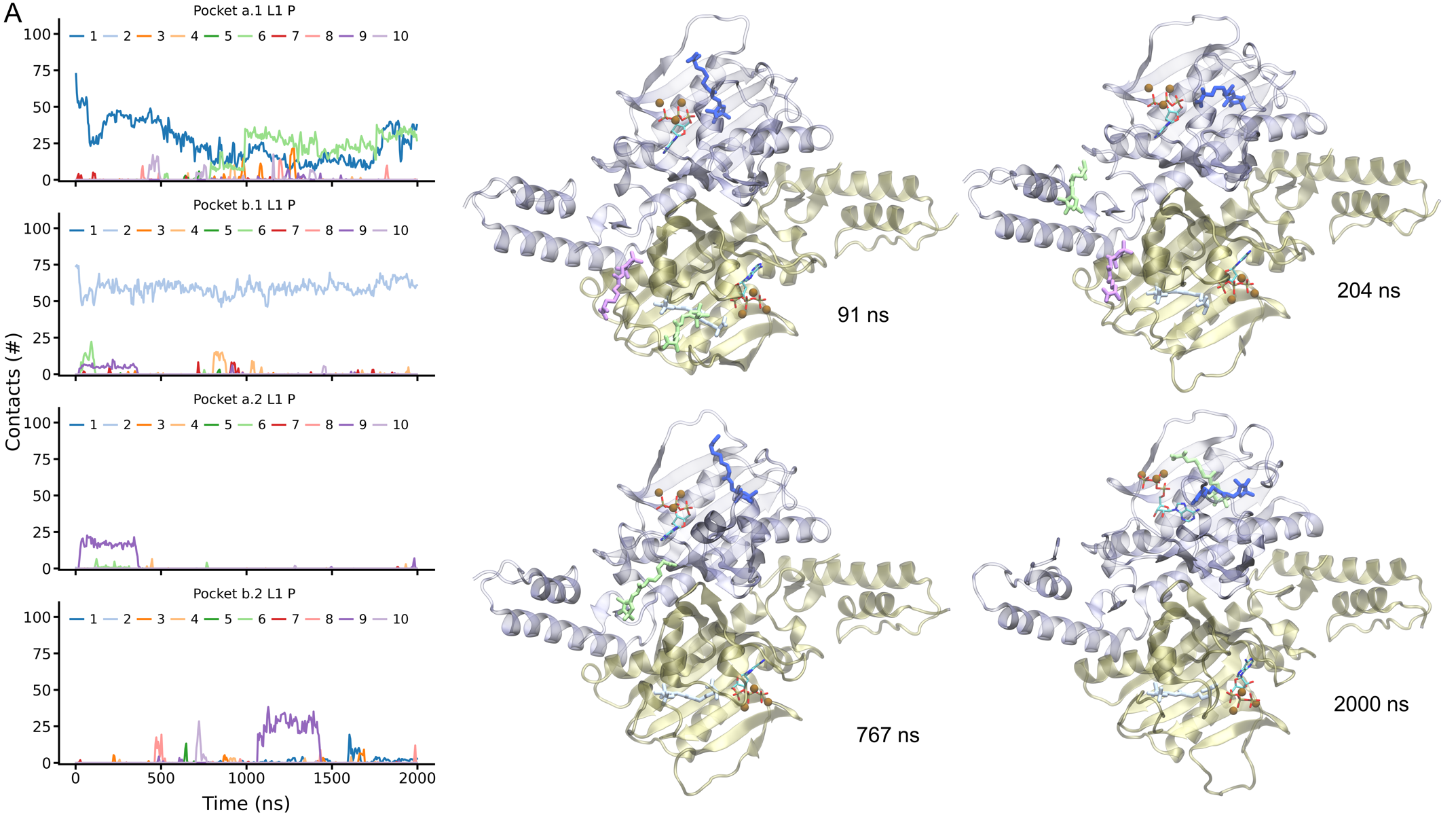


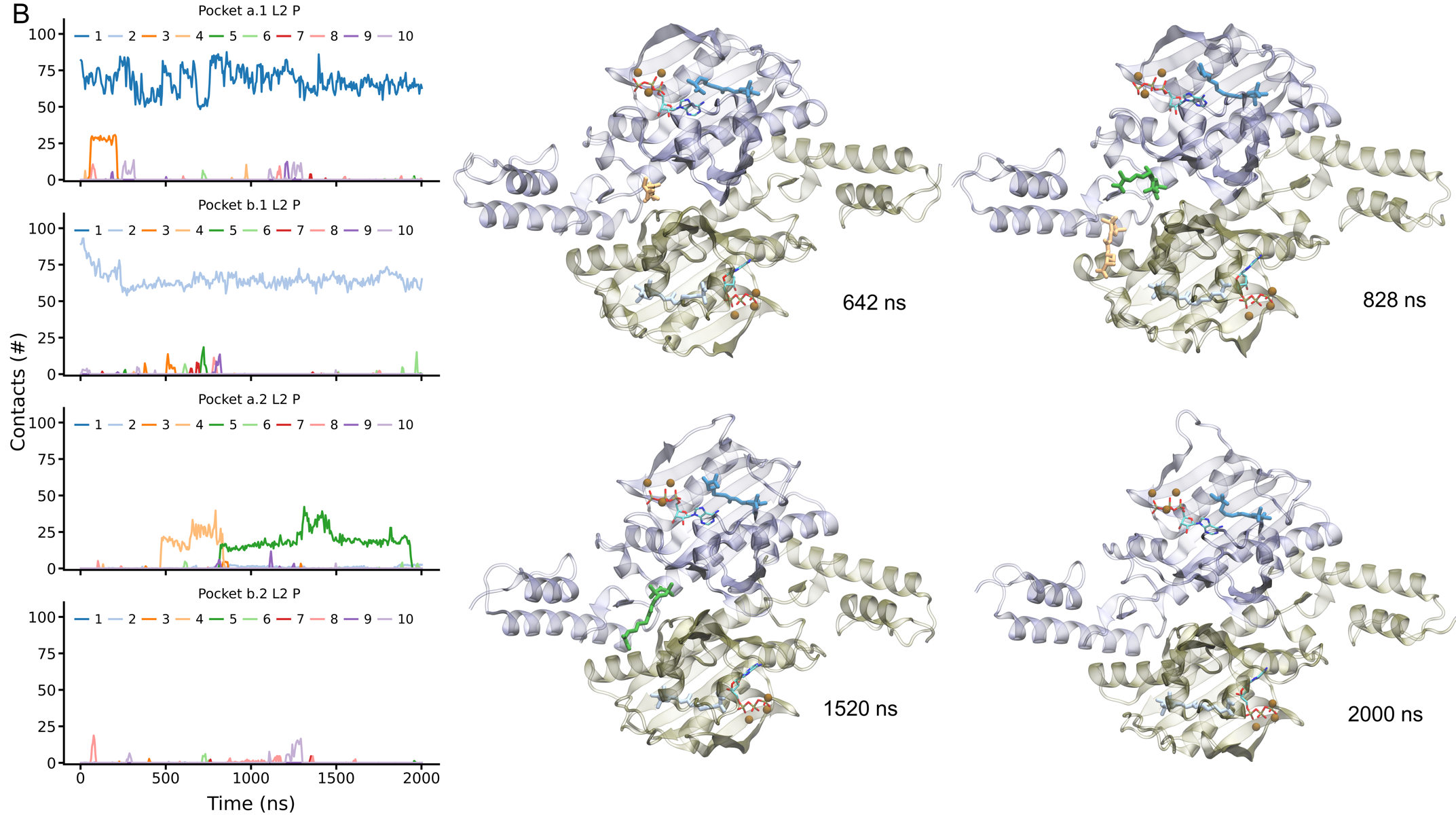


1. Contact analysis for simulations with 8 excess ligands in solution and predocked ligands in both catalytic sites (for protein models L1 (subfigure A) and L2 (subfigure B)). Left column: Contact plots for each ligand binding site (upper two panels: catalytic sites; lower two panels: secondary sites) with lines representing the number of contacts of the individual ligands to the binding sites (with a contact defined as an atom pair with a distance below 0.35 nm). Short double occurrences of one ligand forming contacts to two binding sites are possible as the rims of the secondary pocket are close to the enzymatic pocket. Due to the high concentration of the ligands multiple occupancies of the pockets are possible. Right columns: Exemplary snapshots of the simulations labeled with the corresponding timestamp. The protein is shown in cartoon representation with different colors for protein chains and the ligands in capped sticks representation with colors matching the ligand IDs in the contact plots. The predocked ligands (IDs 1 and 2, i.e. dark blue and light blue lines in pockets a.1 and b.1) remain stably bound to the catalytic binding sites. With the exception of a loosening of ligand 1 in pocket a.1/initial structure L1 where the ligand exhibits an initial reorientation after approximately 60 ns as illustrated in the snapshot at 91 ns and rotates back at around 204 ns. For the free ligands one observes multiple instances where the contact counts go up, i.e. contacts to the catalytic sites or the secondary binding sites are formed that indicate binding events or attempts at binding events. In the case of the secondary binding sites (a.2 and b.2) contacts are formed in all simulations, with a few binding events where the ligand remains in the pocket for time scales of several 100 ns. One example for such a spontaneous binding event can be seen in subfigure B, where the ligands 4 and 5 (orange and green lines) bind successively to pocket a.2. The two snapshots at 828 and 1520 ns nicely illustrate how the ligand rattles around in the binding pocket, yet remains bound for more than 1000 ns.


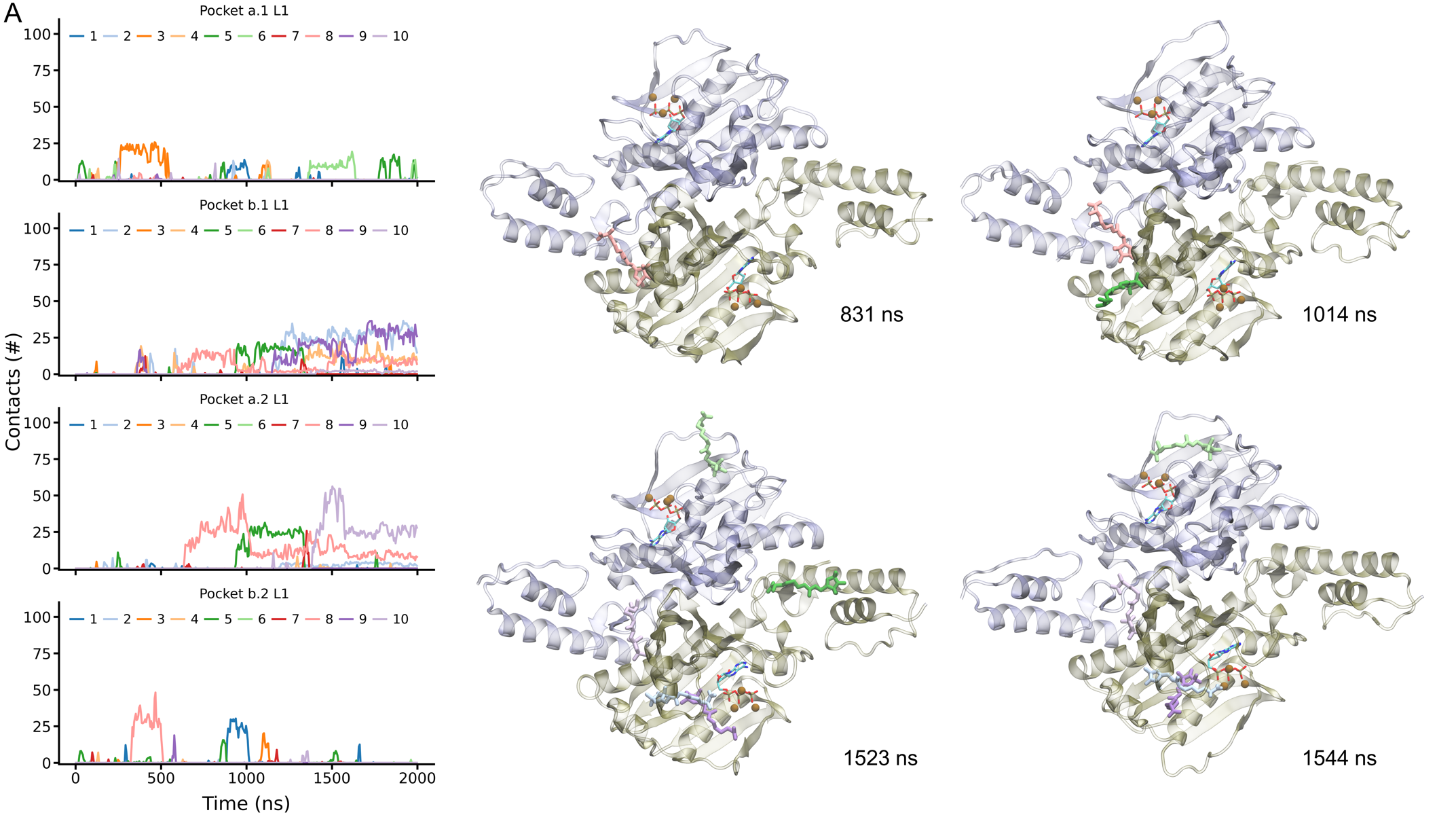


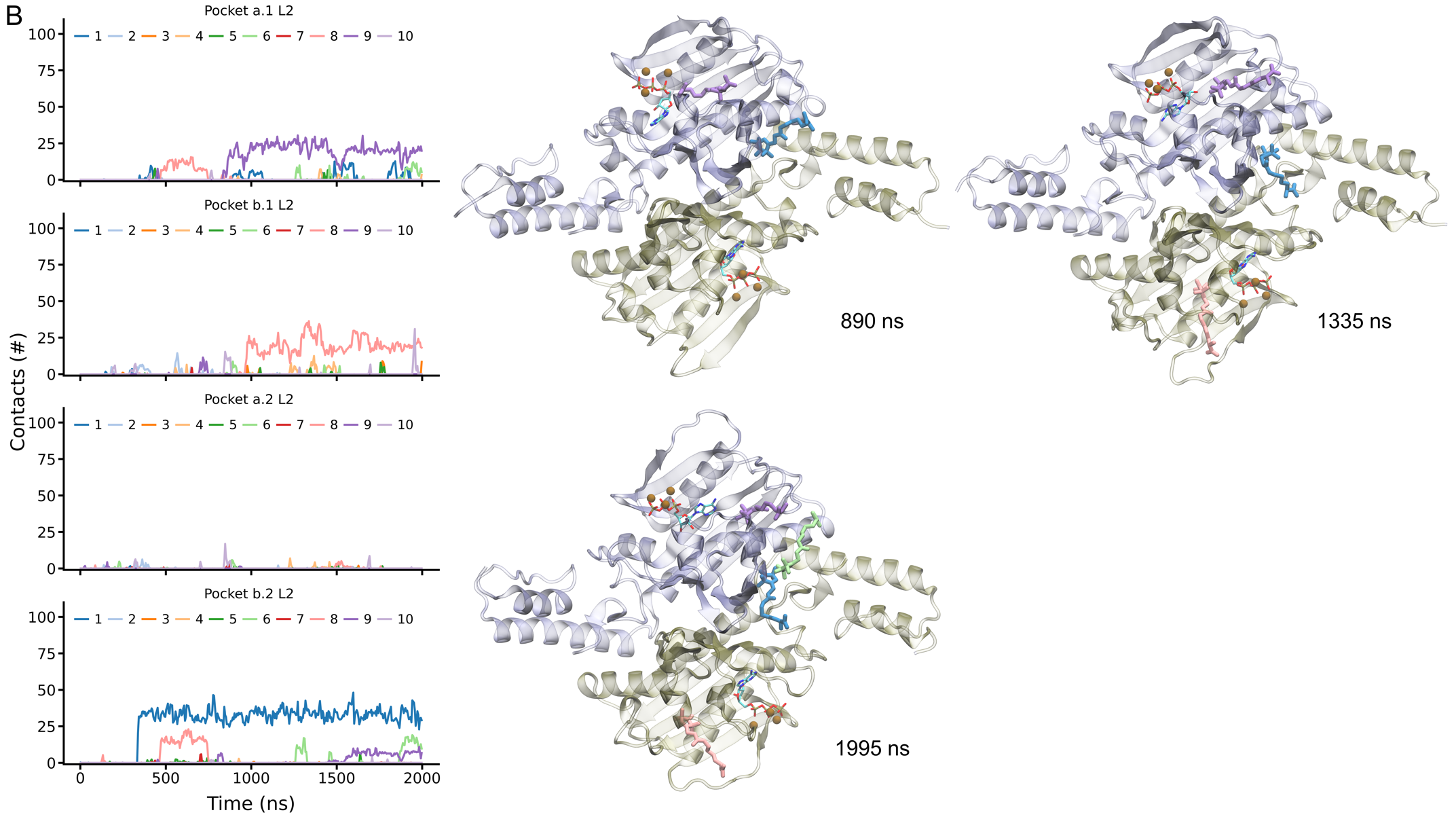


1. Contact analysis for simulations with 10 excess ligands in solution and empty catalytic sites (for protein models L1 (subfigure A) and L2 (subfigure B)). Left column: Contact plots for each ligand binding site (upper two panels: catalytic sites; lower two panels: secondary sites) with lines representing the number of contacts of the individual ligands to the binding sites (with a contact defined as an atom pair with a distance below 0.35 nm). Short double occurrences of one ligand forming contacts to two binding sites are possible as the rims of the secondary pocket are close to the enzymatic pocket. Due to the high concentration of the ligands multiple occupancies of the pockets are possible. Right columns: Exemplary snapshots of the simulations labeled with the corresponding timestamp. The protein is shown in cartoon representation with different colors for protein chains and the ligands in capped sticks representation with colors matching the ligand IDs in the contact plots. After an initial lag phase contact formation with all pockets is observed. For the catalytic pockets, several events occur where a ligand forms long lasting contacts with the binding site, but it should be noted that the binding is less deep (i.e. the number of contacts is lower) compared to the predocked systems. One example can be seen in subfigure B, where the ligands 9 and 8 (purple and pink lines) stay in contact with the entrance of the catalytic binding sites a1 and b1 for more than 1000 ns. The sampling is still too limited to observe a full binding event where one ligand fully slips into the deeper parts of the pocket. This presumably requires a concerted motion of loops building up the pocket entrance (β7-β8 hairpin, Motif 2 loop, Ordering loop^[17]^) – a motion for which we see some indication in the investigation of the conformations of the protein itself, which however will require further analysis and possibly enhanced sampling simulations in the future. For the secondary pockets, extended periods of ligand bindingare observed (see e.g. binding of ligand 1, blue line to binding site b.2 in subfigure B).


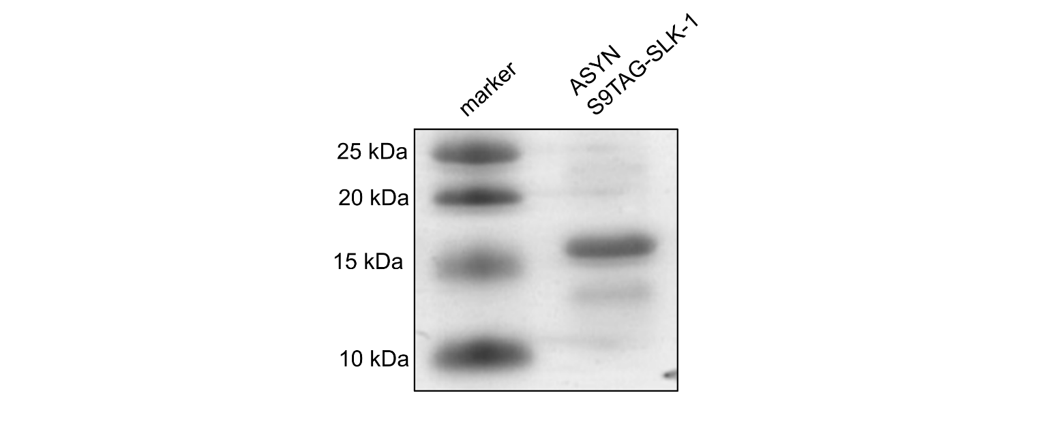


1. Functionality assay of PylRS-SL1 and its cognate tRNA^Pyl^. The tRNA^Pyl^/ PylRS-SL1 pair was expressed in presence of SLK-1 in E. coli cells. SLK-1 was efficiently incorporated in the protein α-synuclein at position 9 in response to the introduced amber stop codon (TAG).


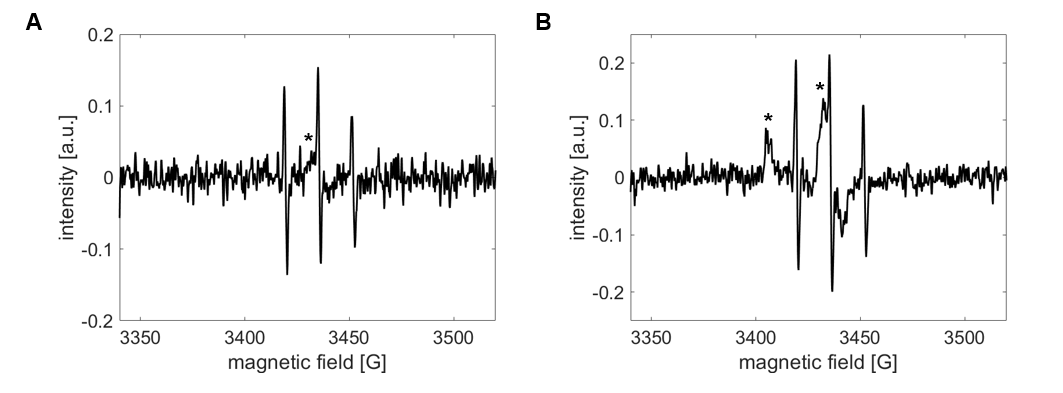


1. In-cell EPR spectra. (A) cdPylRS-SL1 and (B) PylRS-SL1 bound to SLK-1 in-cell. Spectral broadening reflecting SLK-1 binding is indicated by the asterisks.


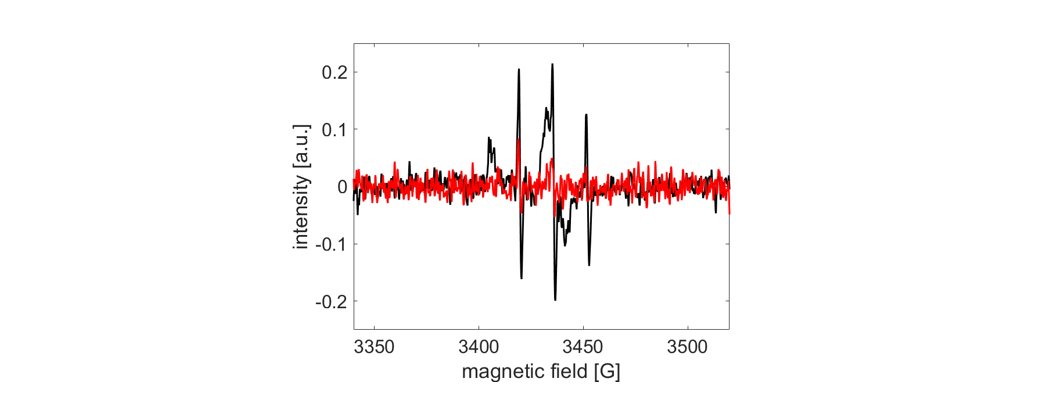


1. SLK-1 does not bind nonspecifically to cellular components. In-cell EPR spectra of PylRS-SL1 bound to SLK-1 (black) compared to cells w/o plasmid that were treated the same way (red). In absence of PylRS-SL1, SLK-1 is removed from the cells and no signal is remaining.


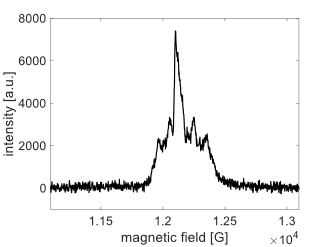


1. In-cell echo-detected field sweep of SLK-1 in presence of cdPylRS-SL1. The nitroxide spectrum overlaps with the spectrum of intracellular Mn(II).


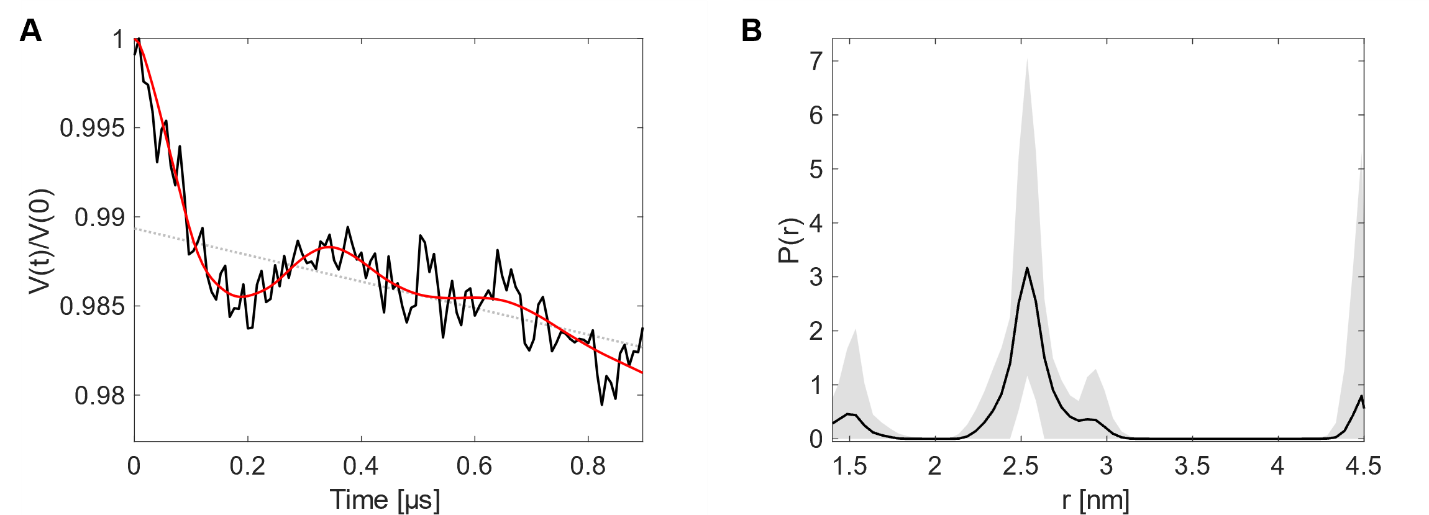


1. In-cell DEER measurement of SLK-1 with cells expressing cdPylRS-SL1. (A) DEER raw data with background (gray) and fit (red). (B) Consensus distance distribution including uncertainty.


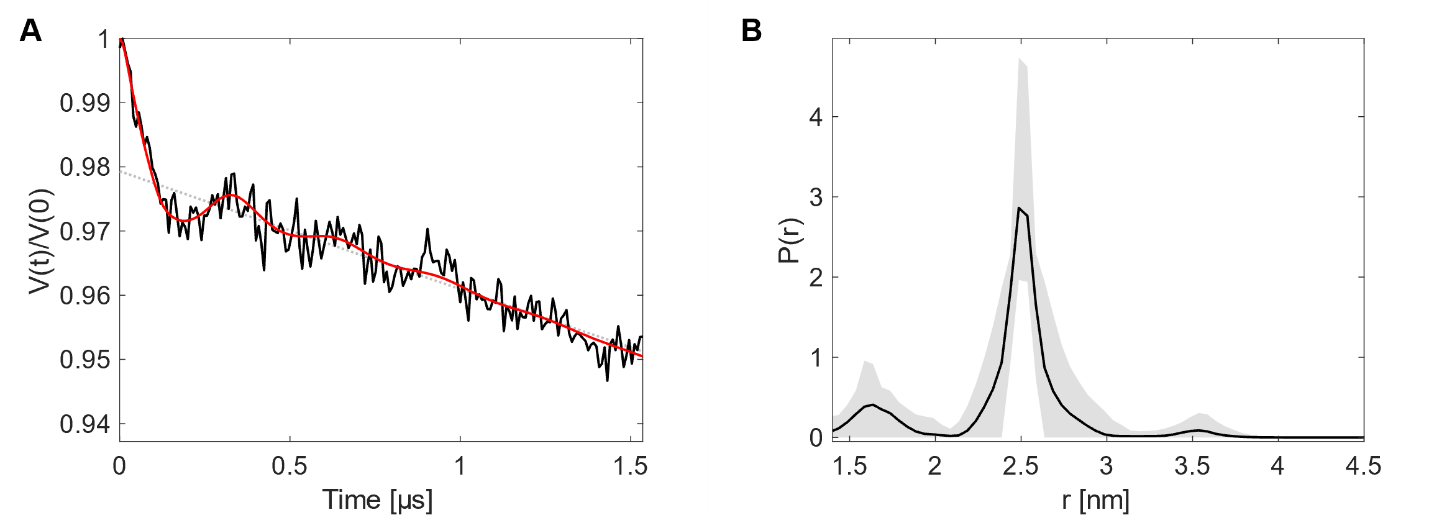


1. In-cell DEER measurement of SLK‑1 with cells expressing PylRS-SL1 (identical sample as in Figure S16). (A) DEER raw data with background (gray) and fit (red). (B) Consensus distance distribution including uncertainty.


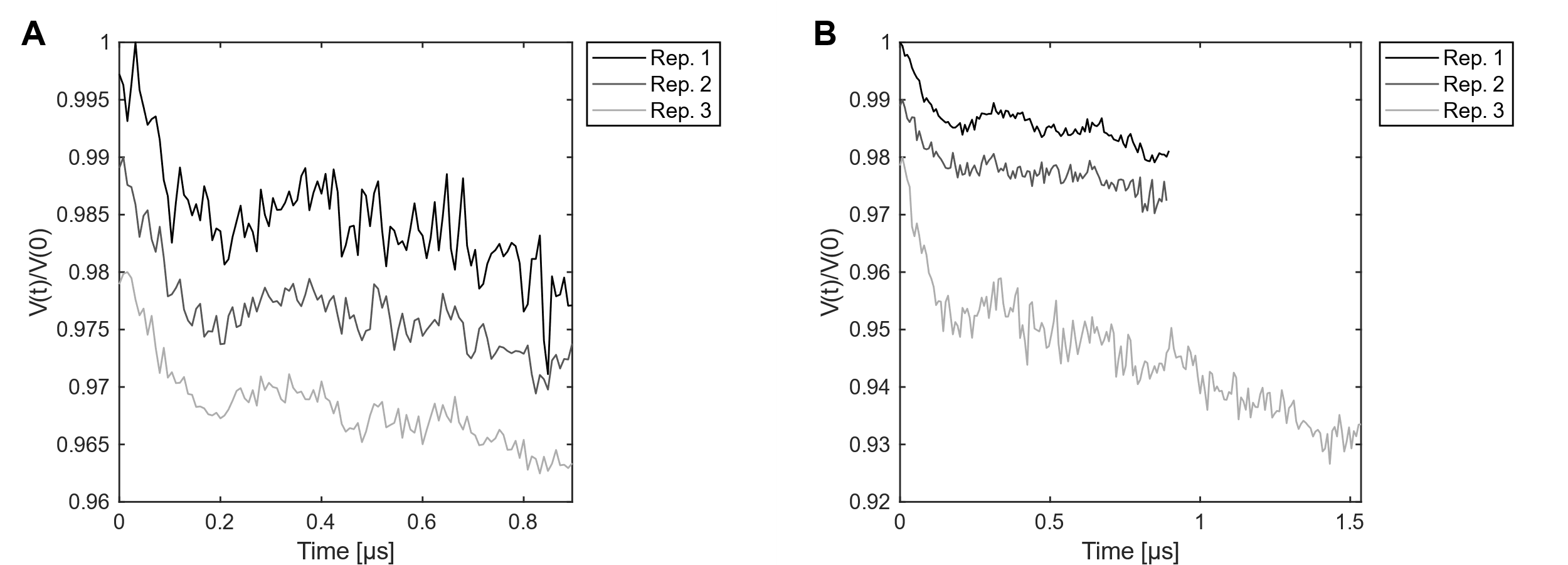


1. DEER raw data of in-cell DEER measurements. (A) Replicates of cdPylRS-SL1. CDA results of Rep. 3 and Rep. 2 are shown in Figures 4 and S16, respectively. For Rep. 1, CDA failed due to poor signal-to-noise ratio. (B) Replicates of PylRS-SL1. CDA results of Rep. 1 and Rep. 3 are shown in Figures 4 and S17, respectively. For Rep. 2, CDA failed due to poor signal-to-noise ratio.


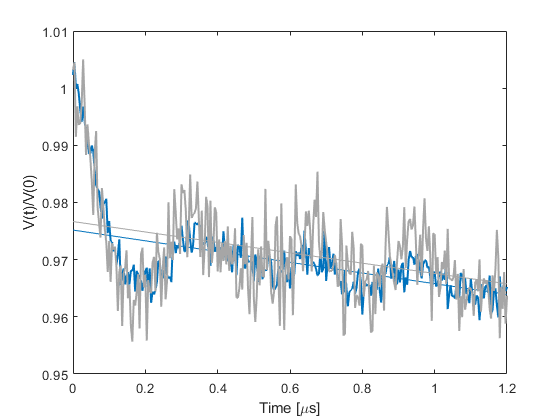


1. Investigating orientation selectivity: DEER measurements of SLK 1 in presence of cdPylRS-SL1 *in vitro*. For both experiments the pump frequency was set to 34.00 GHz and the observer frequency to 33.93 GHz. In order to investigate orientation selection by exciting different parts of the spectrum, the static magnetic field was varied. It was set either to 12092 G (blue) or 12111 G (grey).


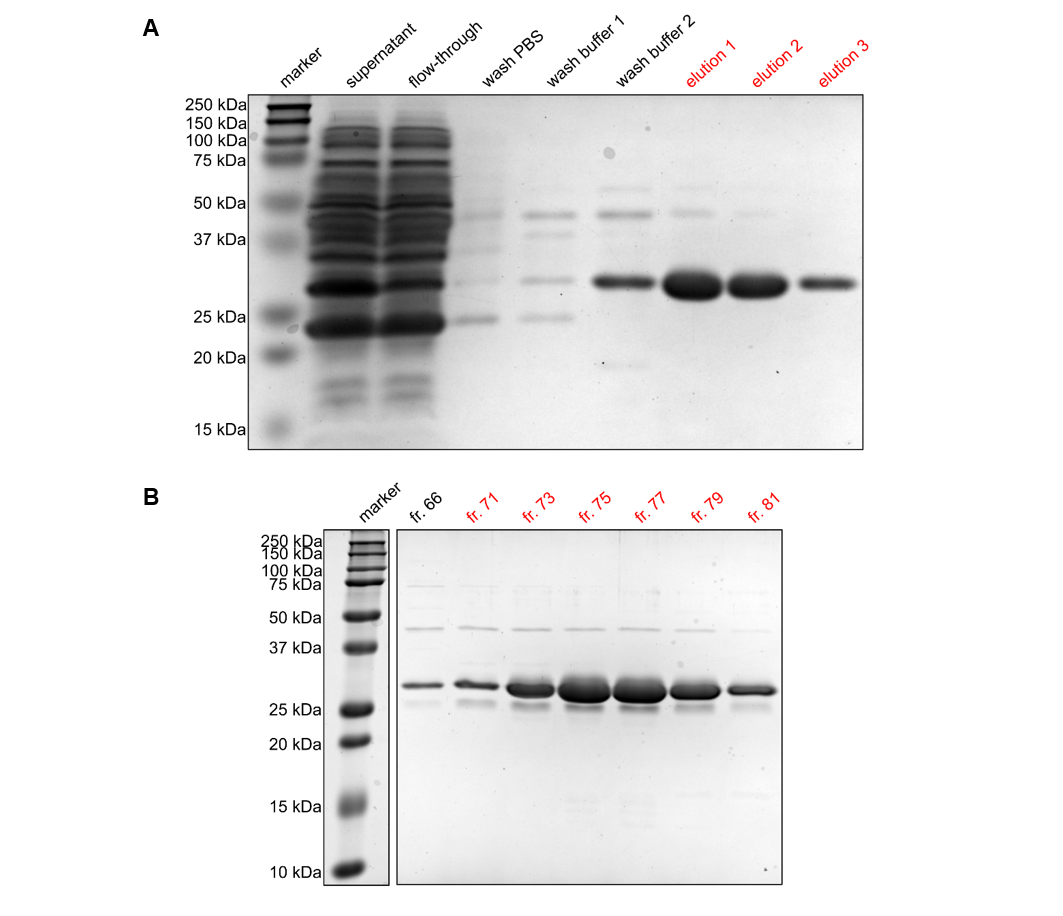


1. Purification of cdPylRS. (A) Purification via His-tag beads. SDS-PAGE shows selected fractions of purification. Elution fractions (red) were taken for further purification. (B) Purification via size exclusion chromatography. SDS-PAGE shows selected fraction of purification. Fractions 70-88 (red) were taken and concentrated.

Seq_1 1 ATGCCAGCACTGACAAAATCCCAAACCGATCGTCTGGAGGTTCTGCTGAATCCGAAAGAC 60

||||||||||||||||||||||||||||||||||||||||||||||||||||||||||||

Seq_2 1 ATGCCAGCACTGACAAAATCCCAAACCGATCGTCTGGAGGTTCTGCTGAATCCGAAAGAC 60

Seq_1 61 GAAATCAGCCTGAATTCCGGCAAACCGTTTCGTGAACTGGAGAGCGAACTGCTGTCACGT 120

||||||||||||||||||||||||||||||||||||||||||||||||||||||||||||

Seq_2 61 GAAATCAGCCTGAATTCCGGCAAACCGTTTCGTGAACTGGAGAGCGAACTGCTGTCACGT 120

Seq_1 121 CGTAAAAAAGACCTGCAACAAATCTATGCCGAAGAACGTGAGAACTATCTGGGGAAACTG 180

||||||||||||||||||||||||||||||||||||||||||||||||||||||||||||

Seq_2 121 CGTAAAAAAGACCTGCAACAAATCTATGCCGAAGAACGTGAGAACTATCTGGGGAAACTG 180

Seq_1 181 GAACGTGAAATCACCCGCTTTTTCGTGGATCGTGGCTTTCTGGAGATCAAATCCCCGATT 240

||||||||||||||||||||||||||||||||||||||||||||||||||||||||||||

Seq_2 181 GAACGTGAAATCACCCGCTTTTTCGTGGATCGTGGCTTTCTGGAGATCAAATCCCCGATT 240

Seq_1 241 CTGATTCCTCTGGAGTATATCGAGCGTATGGGCATCGACAATGATACCGAACTGAGCAAA 300

||||||||||||||||||||||||||||||||||||||||||||||||||||||||||||

Seq_2 241 CTGATTCCTCTGGAGTATATCGAGCGTATGGGCATCGACAATGATACCGAACTGAGCAAA 300

Seq_1 301 CAAATTTTCCGTGTGGATAAAAACTTCTGTCTGCGCCCTATGCTAGCACCAAATCTGGCT 360

||||||||||||||||||||||||||||||||||||||||||||||||||||||||||||

Seq_2 301 CAAATTTTCCGTGTGGATAAAAACTTCTGTCTGCGCCCTATGCTAGCACCAAATCTGGCT 360

Seq_1 361 AACTATCTGCGCAAACTGGACCGTGCCCTGCCTGATCCTATCAAAATCTTCGAGATCGGC 420

||||||||||||||||||||||||||||||||||||||||||||||||||||||||||||

Seq_2 361 AACTATCTGCGCAAACTGGACCGTGCCCTGCCTGATCCTATCAAAATCTTCGAGATCGGC 420

Seq_1 421 CCGTGTTATCGTAAAGAGTCCGACGGTAAAGAACATCTGGAGGAGTTTACCATGCTGAAC 480

||||||||||||||||||||||||||||||||||||||||||||||||||||||||||||

Seq_2 421 CCGTGTTATCGTAAAGAGTCCGACGGTAAAGAACATCTGGAGGAGTTTACCATGCTGAAC 480

Seq_1 481 TTTTGCCAAATGGGTTCAGGTTGTACTCGTGAGAACCTGGAAAGCATCATCACCGATTTT 540

||||||||||||||||||||||||||||||||||||||||||||||||||||||||||||

Seq_2 481 TTTTGCCAAATGGGTTCAGGTTGTACTCGTGAGAACCTGGAAAGCATCATCACCGATTTT 540

Seq_1 541 CTGAACCACCTGGGCATTGACTTCAAAATTGTGGGCGACAGCTGTATGGTGTTTGGCGAC 600

||||||||||||||||||||||||||||||||||||||||||||||||||||||||||||

Seq_2 541 CTGAACCACCTGGGCATTGACTTCAAAATTGTGGGCGACAGCTGTATGGTGTTTGGCGAC 600

Seq_1 601 ACCCTGGATGTCATGCACGGCGACCTGGAACTGTCTAGTGCCGTTGTTGGACCAATTCCG 660

||||||||||||||||||||||||||||||||||||||||||||||||||||||||||||

Seq_2 601 ACCCTGGATGTCATGCACGGCGACCTGGAACTGTCTAGTGCCGTTGTTGGACCAATTCCG 660

Seq_1 661 CTGGACCGTGAGTGGGGTATCGACAAACCGTGGATCGGAGCAGGATTCGGTCTGGAACGC 720

|||||||||||||||||| | |||||||||||||||||||||||||||||||||||||||

Seq_2 661 CTGGACCGTGAGTGGGGTCTGGACAAACCGTGGATCGGAGCAGGATTCGGTCTGGAACGC 720

Seq_1 721 CTGCTGAAAGTGAAACACGACTTCAAAAACATCAAACGTGCCGCCCGTTCTGAATCGTAT 780

||||||||||||||||||||||||||||||||||||||||||||||||||||||||||||

Seq_2 721 CTGCTGAAAGTGAAACACGACTTCAAAAACATCAAACGTGCCGCCCGTTCTGAATCGTAT 780

Seq_1 781 TATAACGGGATCTCTACGAACCTGCATCATCATCATCATCATTGA 825

|||||||||||||||||||||||| | |

Seq_2 781 TATAACGGGATCTCTACGAACCTG--TAA---------------- 807

1. Sequence alignment of cdPylRS (gray, Seq 1) and cdPylRS-SL1 (black, Seq 2). The position of the I to L mutation is shown in pink. The His_6_-tag is highlighted in blue and the stop codons are colored in red. Instead of the His_6_-tag, plasmid cdPylRS-SL1 exhibits a stop codon (TAA, red).


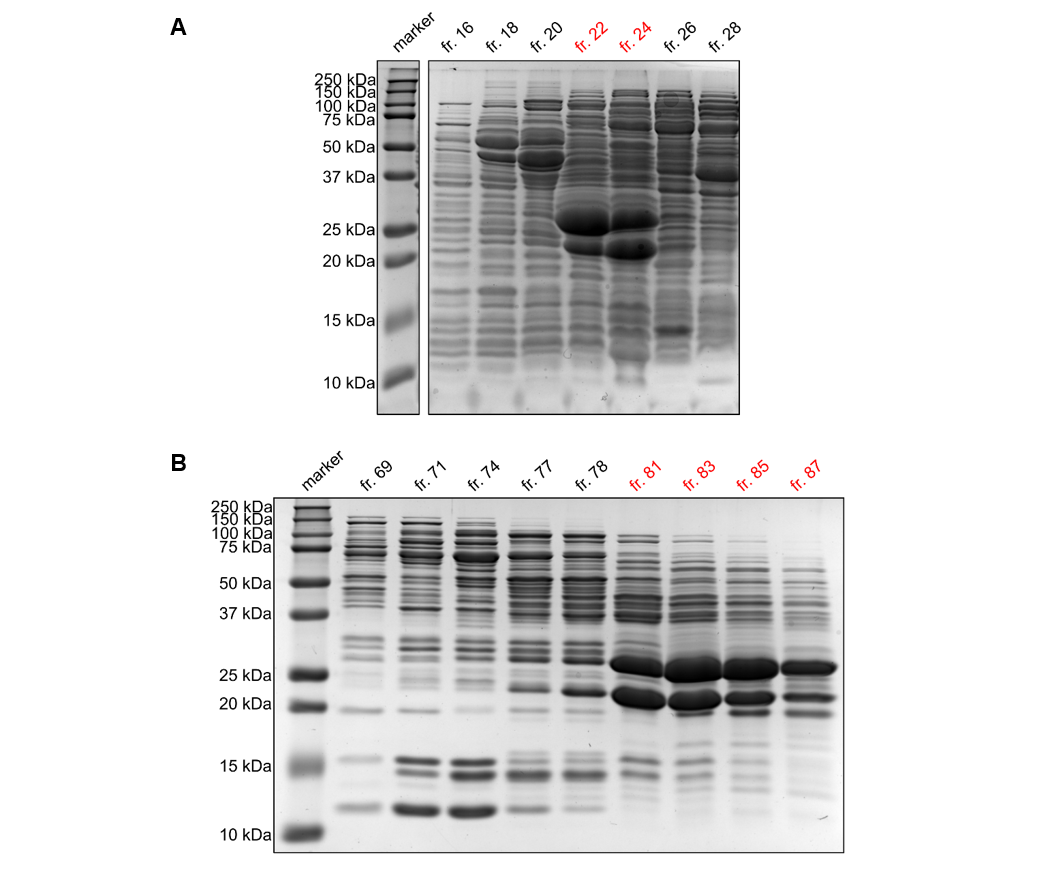


1. Purification of cdPylRS-SL1. (A) Purification via anion exchange chromatography. SDS-PAGE shows selected fractions of purification. Fractions 22-24 (red) were taken for further purification. (B) Purification via size exclusion chromatography. SDS-PAGE shows selected fraction of purification. Fractions 80-88 (red) were taken and concentrated.

# References

[1] M. J. Schmidt, A. Weber, M. Pott, W. Welte, D. Summerer, *ChemBioChem* **2014**, *15*, 1755–1760.

[2] M. J. Schmidt, J. Borbas, M. Drescher, D. Summerer, *J. Am. Chem. Soc.* **2014**, *3*, 1–33.

[3] S. Stoll, A. Schweiger, *J. Magn. Reson.* **2006**, *178*, 42–55.

[4] M. Pannier, S. Veit, A. Godt, G. Jeschke, H. W. Spiess, *J. Magn. Reson.* **2000**, *142*, 331–340.

[5] C. E. Tait, S. Stoll, *Phys. Chem. Chem. Phys.* **2016**, *18*, 18470–18485.

[6] G. Jeschke, V. Chechik, P. Ionita, A. Godt, H. Zimmermann, J. Banham, C. R. Timmel, D. Hilger, H. Jung, *Appl. Magn. Reson.* **2006**, *30*, 473–498.

[7] M. J. Schmidt, A. Fedoseev, D. Summerer, M. Drescher, *Methods Enzymol.* **2015**, *563*, 483–502.

[8] S. Steigmiller, M. Börsch, P. Gräber, M. Huber, *Biochim. Biophys. Acta - Bioenerg.* **2005**, *1708*, 143–153.

[9] A. Gamble Jarvi, J. Casto, S. Saxena, *J. Magn. Reson.* **2020**, *320*, 106848.

[10] A. Scherer, S. Tischlik, S. Weickert, V. Wittmann, M. Drescher, *Magn. Reson.* **2020**, *1*, 59–74.

[11] T. Yanagisawa, M. Kuratani, E. Seki, N. Hino, K. Sakamoto, S. Yokoyama, *Cell Chem. Biol.* **2019**, *26*, 936–949.

[12] M. Ragoza, J. Hochuli, E. Idrobo, J. Sunseri, D. R. Koes, *J. Chem. Inf. Model.* **2017**, *57*, 942–957.

[13] A. T. McNutt, P. Francoeur, R. Aggarwal, T. Masuda, R. Meli, M. Ragoza, J. Sunseri, D. R. Koes, *J. Cheminform.* **2021**, *13*, 1–20.

[14] G. M. Morris, H. Ruth, W. Lindstrom, M. F. Sanner, R. K. Belew, D. S. Goodsell, A. J. Olson, *J. Comput. Chem.* **2009**, *30*, 2785–2791.

[15] X. Daura, K. Gademann, B. Jaun, D. Seebach, W. F. van Gunsteren, A. E. Mark, A. Rigault, J. Siegel, J. Harrowfield, B. Chevrier, D. Moras, J. Lehn, M. Garrett, U. Koert, D. Meyer, J. Fischer, *Angew. Chemie Int. Ed.* **1998**, *31*, 1387–1404.

[16] M. J. Abraham, T. Murtola, R. Schulz, S. Páll, J. C. Smith, B. Hess, E. Lindah, *SoftwareX* **2015**, *1*–*2*, 19–25.

[17] S. Pronk, S. Páll, R. Schulz, P. Larsson, P. Bjelkmar, R. Apostolov, M. R. Shirts, J. C. Smith, P. M. Kasson, D. Van Der Spoel, B. Hess, E. Lindahl, *Bioinformatics* **2013**, *29*, 845–854.

[18] K. Vanommeslaeghe, A. D. MacKerell, *J. Chem. Inf. Model.* **2012**, *52*, 3144–3154.

[19] K. Vanommeslaeghe, E. P. Raman, A. D. MacKerell, *J. Chem. Inf. Model.* **2012**, *52*, 3155–3168.

[20] B. R. Brooks, C. L. Brooks, A. D. Mackerell, L. Nilsson, R. J. Petrella, B. Roux, Y. Won, G. Archontis, C. Bartels, S. Boresch, A. Caflisch, L. Caves, Q. Cui, A. R. Dinner, M. Feig, S. Fischer, J. Gao, M. Hodoscek, W. Im, K. Kuczera, T. Lazaridis, J. Ma, V. Ovchinnikov, E. Paci, R. W. Pastor, C. B. Post, J. Z. Pu, M. Schaefer, B. Tidor, R. M. Venable, H. L. Woodcock, X. Wu, W. Yang, D. M. York, M. Karplus, *J. Comput. Chem.* **2009**, *30*, 1545–1614.

[21] J. Huang, S. Rauscher, G. Nawrocki, T. Ran, M. Feig, B. L. De Groot, H. Grubmüller, A. D. MacKerell, *Nat. Methods* **2016**, *14*, 71–73.

[22] E. Lindahl, P. Bjelkmar, P. Larsson, M. A. Cuendet, B. Hess, *J. Chem. Theory Comput.* **2010**, *6*, 459–466.

[23] S. Jo, T. Kim, V. G. Iyer, W. Im, *J. Comput. Chem.* **2008**, *29*, 1859–1865.

[24] J. Lee, X. Cheng, J. M. Swails, M. S. Yeom, P. K. Eastman, J. A. Lemkul, S. Wei, J. Buckner, J. C. Jeong, Y. Qi, S. Jo, V. S. Pande, D. A. Case, C. L. Brooks, A. D. MacKerell, J. B. Klauda, W. Im, *J. Chem. Theory Comput.* **2016**, *12*, 405–413.

[25] S. Kim, J. Lee, S. Jo, C. L. Brooks, H. S. Lee, W. Im, *J. Comput. Chem.* **2017**, *38*, 1879–1886.

[26] W. F. Van Gunsteren, H. J. C. Berendsen, *Mol. Simul.* **2007**, *1*, 173–185.

[27] B. Hess, H. Bekker, H. J. C. Berendsen, J. G. E. M. Fraaije, *J. Comput. Chem.* **1997**, *18*, 14631472.

[28] U. Essmann, L. Perera, M. L. Berkowitz, T. Darden, H. Lee, L. G. Pedersen, *J. Chem. Phys.* **1998**, *103*, 8577.

[29] S. Nosé, *Mol. Phys.* **1984**, *52*, 255–268.

[30] W. G. Hoover, *Phys. Rev. A* **1985**, *31*, 1695.

[31] M. Parrinello, A. Rahman, *J. Appl. Phys.* **1981**, *52*, 7182.

[32] E. F. Pettersen, T. D. Goddard, C. C. Huang, G. S. Couch, D. M. Greenblatt, E. C. Meng, T. E. Ferrin, *J. Comput. Chem.* **2004**, *25*, 1605–1612.

[33] T. D. Goddard, C. C. Huang, E. C. Meng, E. F. Pettersen, G. S. Couch, J. H. Morris, T. E. Ferrin, *Protein Sci.* **2018**, *27*, 14–25.

[34] E. F. Pettersen, T. D. Goddard, C. C. Huang, E. C. Meng, G. S. Couch, T. I. Croll, J. H. Morris, T. E. Ferrin, *Protein Sci.* **2021**, *30*, 70–82.

[35] W. Humphrey, A. Dalke, K. Schulten, *J. Mol. Graph.* **1996**, *14*, 33–38.
